# Multifunctional bioactive glass nanoparticles: surface–interface decoration and biomedical applications

**DOI:** 10.1093/rb/rbae110

**Published:** 2024-09-06

**Authors:** Mi Chen, Yidan Wang, Pingyun Yuan, Lan Wang, Xiaocheng Li, Bo Lei

**Affiliations:** Shaanxi Key Laboratory of Biomedical Metallic Materials, Northwest Institute for Non-Ferrous Metal Research, Xi'an 710016, China; Shaanxi Key Laboratory of Biomedical Metallic Materials, Northwest Institute for Non-Ferrous Metal Research, Xi'an 710016, China; Shaanxi Key Laboratory of Biomedical Metallic Materials, Northwest Institute for Non-Ferrous Metal Research, Xi'an 710016, China; Shaanxi Key Laboratory of Biomedical Metallic Materials, Northwest Institute for Non-Ferrous Metal Research, Xi'an 710016, China; Shaanxi Key Laboratory of Biomedical Metallic Materials, Northwest Institute for Non-Ferrous Metal Research, Xi'an 710016, China; Key Laboratory of Shaanxi Province for Craniofacial Precision Medicine Research, College of Stomatology, Xi'an Jiaotong University, Xi’an 710000, China; Frontier Institute of Science and Technology, Xi’an Jiaotong University, Xi’an 710000, China

**Keywords:** bioactive glass nanoparticles, surface modification, functionalization, multifunctional properties

## Abstract

Developing bioactive materials with multifunctional properties is crucial for enhancing their biomedical applications in regenerative medicine. Bioactive glass nanoparticle (BGN) is a new generation of biomaterials that demonstrate high biocompatibility and tissue-inducing capacity. However, the hard nanoparticle surface and single surface property limited their wide biomedical applications. In recent years, the surface functional strategy has been employed to decorate the BGN and improve its biomedical applications in bone tissue repair, bioimaging, tumor therapy and wound repair. This review summarizes the progress of surface–interface design strategy, customized multifunctional properties and biomedical applications in detail. We also discussed the current challenges and further development of multifunctional BGN to meet the requirements of various biomedical applications.

## Graphical abstract

**Figure:**
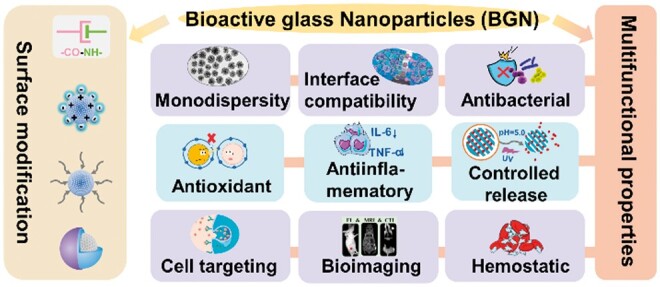

## Introduction

In 1969, Professor Larry L. Hench developed the first generation of bioactive glass (BG, 46.1SiO_2_-2.6P_2_O_5_-24.4Na_2_O-26.9CaO) [1, 2]. It is a kind of material that can repair, replace, and regenerate body tissues and form bonds between tissues and materials. It not only has osteogenesis but also osteoinduction [3]. After Professor Hench had developed the bioactive glass for the first time, the upsurge of BG started. In 1974, Professor Brömer prepared microcrystalline bioglass (Ceravital glass) by adjusting the content of alkali metal oxides. Its bioactivity is lower than conventional 45S5 glass, but its mechanical properties have been strikingly augmented, which can be used to fill bone defects with less obvious stress [4]. In addition, Professor Kokubo used heat treatment technology to prepare high-strength MgO-CaO-SiO_2_-P_2_O_5_-CaF_2_ bioactive glass ceramics in 1982. The glass matrix contains crystalline apatite and β-Wollastonite and is currently the best medical glass-ceramic material with relatively strong mechanical properties. In addition, its bonding strength with bone tissue is also high [5]. In the same period, Holland successfully developed machinable biological glass ceramics with the trade name of ‘Bioverit’, which has both specific biological activity and outstanding machinability and can be rapidly processed according to clinical needs [6]. With the deepening of studies, the synthesis of BG has made a breakthrough. Some commercial BG materials have been certified by the Food and Drug Administration (FDA) and the European Committee for Standardization (CEN), which can be used in bone repair and replacement [7]. In 1991, Professor Hench introduced the sol-gel technology into the synthesis of BG, which transformed BG from a completely dense structure into a porous material, and the composition of BG was controlled, which provided a new opportunity for the broad application of BG [8, 9].In 2004, the group of Professor Dongyuan Zhao prepared highly ordered mesoporous bioactive glass for the first time by adopting the sol-gel method combined with template assembly technology and selecting the triblock copolymer P123 as the template. Compared with the conventional sol-gel bioactive glass, the former has a more ordered mesoporous structure, a larger specific surface area, and a faster apatite formation activity [10].

**Figure 1.**
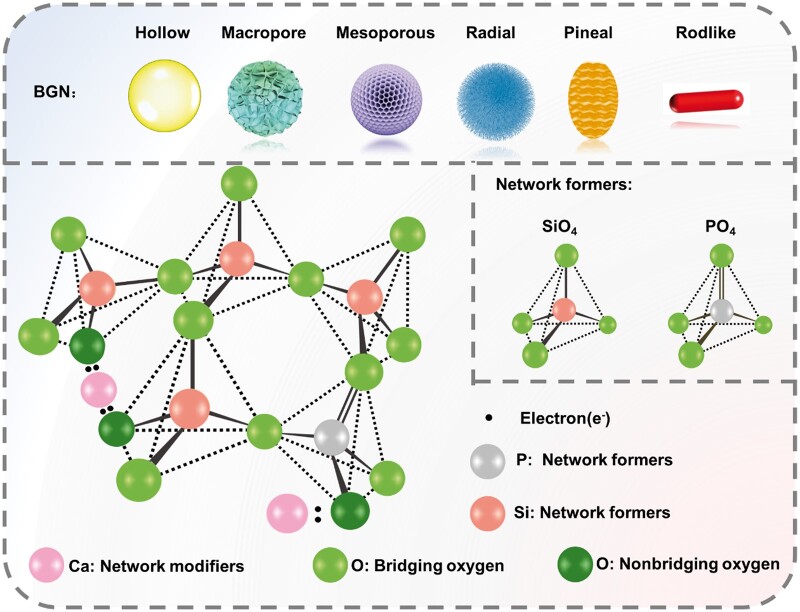
Morphology and molecular structure of BGN.

In addition, the group of Professor Xiaofeng Chen has developed BGN using a sol-gel method combined with molecular template technology, which significantly expands the function and bioactivity of BG material [11–13]. Before that, BG was applied in block form. The emergence of nanotechnology has brought new vitality to the application of biomedicine. At present, the preparation methods of BGN mainly include the flame jet, microemulsion, and sol-gel methods. BGN with different morphologies can be prepared by adjusting the synthesis process, such as hollow, macroporous, mesoporous, radial, pineal, and rod-shaped [14–19]. The molecular structure of BGN is the same as that of conventional BG. It is usually composed of three components: network formers, network modifiers and intermediate oxides (Figure 1) [20]. It is worth mentioning that although it is generally accepted that nanomaterials mean that at least one of the three-dimensional dimensions of materials is in the range of 1–100 nm, the size of most reported BGN is actually in the sub-micron scale (100–1000 nm), which is collectively referred to as BGN.

In addition to the inherent properties of BG, such as biological activity, bone induction, biocompatibility and degradability, BGN also has a passive-targeting and drug-loading capacity (drugs, proteins or genes) different from BG to achieve disease treatment [7, 21–25]. In recent years, many scholars at home and abroad have conducted extensive and in-depth research on BGN, accelerating the application of BGN in hard tissue repair, soft tissue repair, disease diagnosis and treatment [26–31]. A common method of using BGN in hard tissue applications is to combine it with biodegradable polymers to form composite materials. These biodegradable polymers include natural polymers such as collagen [32], sodium alginate [33], chitin [34], chitosan [35], hyaluronic acid [36], gelatin [37], gellan gum [38], silk fibroin [39], as well as synthetic polymers such as poly (3-hydroxybutyrate) (P(3HB)) [40], poly (L-lactic acid) (PLLA) [41], poly (d, l-lactide) [42], and poly citric acid (PC) [43]. These composite materials aim to impart strength and biological activity through inorganic bioactive fillers while maintaining the favorable properties of the polymer, such as flexibility and the ability to deform under load. In addition, some teams have 3D printed them to form specific scaffolds to adapt to complex bone defect environments [44–46]. In the field of soft tissue repair, Professor Xiaofeng Chen’s team has confirmed that BGN in the traditional Si/Ca system has a specific regulatory effect on macrophages, endothelial cells, fibroblasts, myofibroblasts, and keratinocytes during wound repair [47–50]. In addition, Professor Aldo R. Boccaccini’s team has confirmed that BGN can promote the proliferation of human skin fibroblasts and human keratinocytes and wound hemostasis [51]. The team of Professor Jiang Chang and Professor Chengtie Wu found that under the high glucose environment *in vitro*, the bioceramics containing silicon ions can promote the proliferation and migration of vascular endothelial cells and accelerate the healing of diabetes wounds [52]. This study provides a strong theoretical foundation for expanding the application of BGN. In the field of disease diagnosis and treatment, BGN can be used as an anti-tumor drug carrier for tumor treatment based on its nanoscale effect and mesoporous structure. In addition, its unique passive targeting effect can further improve the therapeutic effect of tumors. And the tumor imaging effect of BGN can be achieved through ion doping [53]. Therefore, BGN has excellent potential for application in tumor therapy. Yazdanpanah *et al.* prepared iron/lithium doped BGN through the sol-gel technology. The BGN can provide a temperature of 47.2°C at most for cancer hyperthermia [54]. Liu *et al.* fully utilized the easy doping and mesoporous properties of BGN to achieve synergistic therapy (photodynamic and photothermal therapy) for bone tumors [55]. In addition, Bernardeschi *et al.* found that BGN has extraordinary radiological manifestations that can be detected by magnetic resonance imaging (MRI) technology for cholesteatoma and/or other potential complications. At the same time, in high-resolution computed tomography (HRCT) scans, the degree of attenuation of BGN in different tissues can be observed, which is expected to achieve the CT imaging function of BGN [56].

However, many issues still need to be addressed regarding the research and widespread application of BGN’s multifunctional properties. First of all, for the application mode of BGN, it is usually used as a filling material to prepare a series of biological composite scaffolds through simple composite blending with natural or synthetic polymer materials such as collagen, chitosan, gelatin, polylactic acid, polycaprolactone, etc., which are used in the field of bone tissue engineering and soft tissue repair. However, due to the differences in the hydrophilicity and hydrophobicity between the BGN inorganic phase and the polymer organic phase, there is no good bonding interface between the two phases. The simple physical packaging and mixing between the polymer and inorganic phases will lead to low bonding strength between the two phases and even the stripping of BGN particles, which will affect the strength of the composite scaffold [57]. Secondly, for the nano-size effect of BGN, on the one hand, the nano-size structure endows BGN with a high specific surface area and surface energy, which is conducive to drug loading. On the other hand, nanomaterial surface reactivity also makes it easier for BGN to form aggregates in the body fluid environment, which hinders its circulation in the body and is not conducive to drug transport, limiting the application of BGN in drug carriers [58]. Then, for the biocompatibility of BGN, although BGN shows superior biocompatibility to cells, studies have found that when a higher concentration of BGN contacts with red blood cells, it will cause the rupture of red blood cells, showing a severe hemolysis phenomenon [53]. Moreover, the release of ions in BGN will cause the pH value of local tissues to rise to 8–11, thus causing tissue inflammation or cytotoxicity [59]. Finally, for the functionality of BGN, the main reason for BGN to play the role of treatment and repair at present is the inherent biological activity, degradation properties and nanoscale effects of BGN, but this cannot meet the requirements of tissue repair in clinical multi-pathological environments. For example, for the treatment of chronic diabetic wounds, it is necessary to solve the inflammatory environment and excessive oxidative stress in the wounds and then pay attention to preventing bacterial infection of the wounds [47, 60]. In addition, the overexpression of inflammatory factors in the tumor microenvironment and the potential tumor recurrence behavior made it difficult for the wound to heal normally [61]. Therefore, bioactive materials with anti-inflammatory, anti-tumor and anti-recurrence effects can solve the above problems effectively. Therefore, it is significant to improve the monodispersity, blood compatibility and multifunctional properties of BGN by surface modification (m-BGN). However, most existing reviews focus on BG or BGN composites and their biomedical applications [57, 62–64]. There are few specific reviews on the surface modification of BGN. Therefore, this review systematically summarizes the methods and functions of BGN surface modification.

## Surface–interface decoration strategy

**Figure 2.**
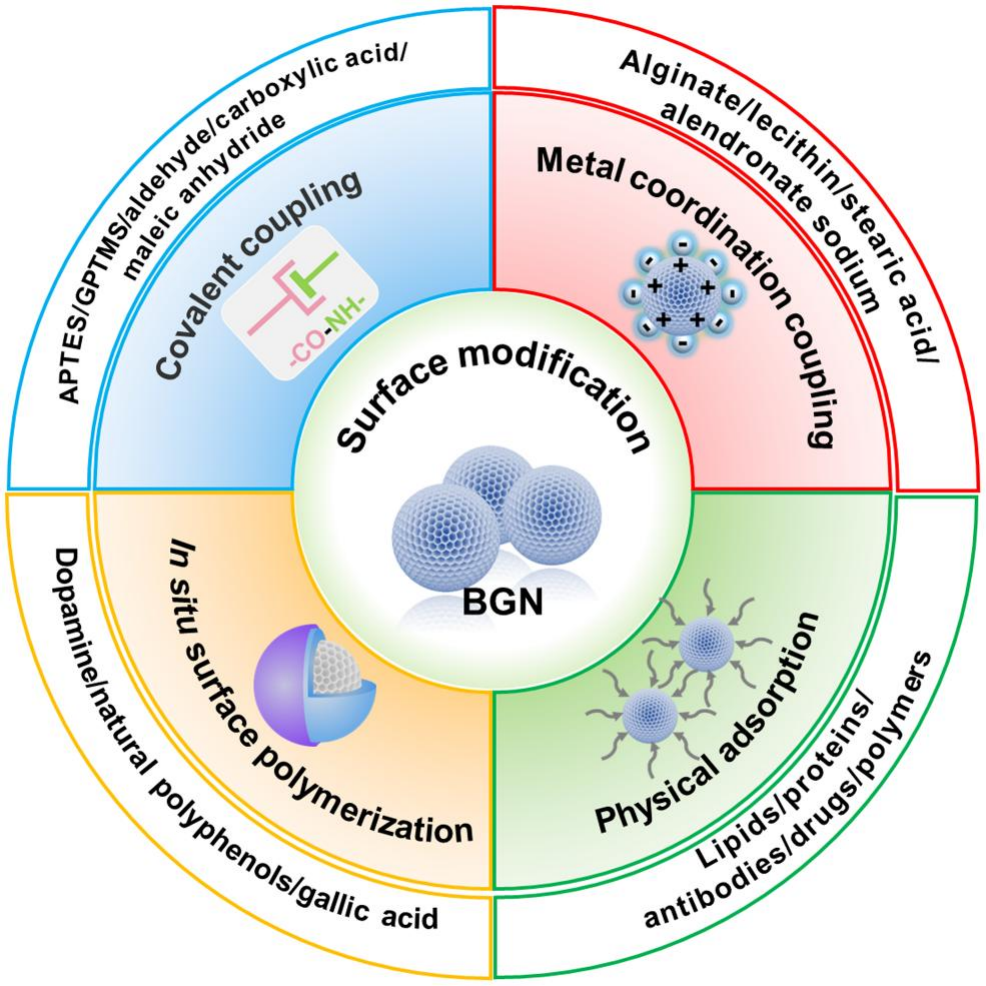
Surface modification technology for BGN.

According to the inherent surface structure and properties of BGN, different strategies and methods can be used to functionalize it, as shown in Figure 2. First, the surface of BGN contains residual Si-OH groups. Therefore, Si-OH can be used as the reactive active site. On the one hand, Si-OH reacts directly with the active molecule containing -COOH; on the other hand, Si-OH reacts with various silane coupling agents to hydrolyze and polymerize, thus introducing other active groups to the surface of BGN [65]. This surface modification method of BGN by chemical reaction is collectively called the covalent coupling method. Secondly, various metal ions contained in the structural composition of BGN, such as Ca^2+^, Fe^3+^, Cu^2+^and Ag^+^, can combine with phosphate and carboxylate through ionic bond interaction so that bioactive materials containing phosphate or carboxylate can be modified on the surface of BGN [66], which is collectively referred to as metal coordination coupling. In addition, the nanostructure of BGN makes it have a large specific surface area, and BGN presents a mesoporous structure, which can adsorb small molecule drugs or compounds to the surface of BGN to achieve the purpose of surface modification [67]. This surface modification method is called physical adsorption. Finally, the BGN surface is utilized as the substrate layer, enabling the monomer to undergo *in situ* oxidative polymerization on its surface directly. This process generates a layer of bioactive coating, thereby achieving the objective of multifunctional modification of the BGN surface [68]. An *in situ* modification strategy without reacting with BGN groups is *in situ* surface polymerization.

### Covalent coupling

**Figure 3.**
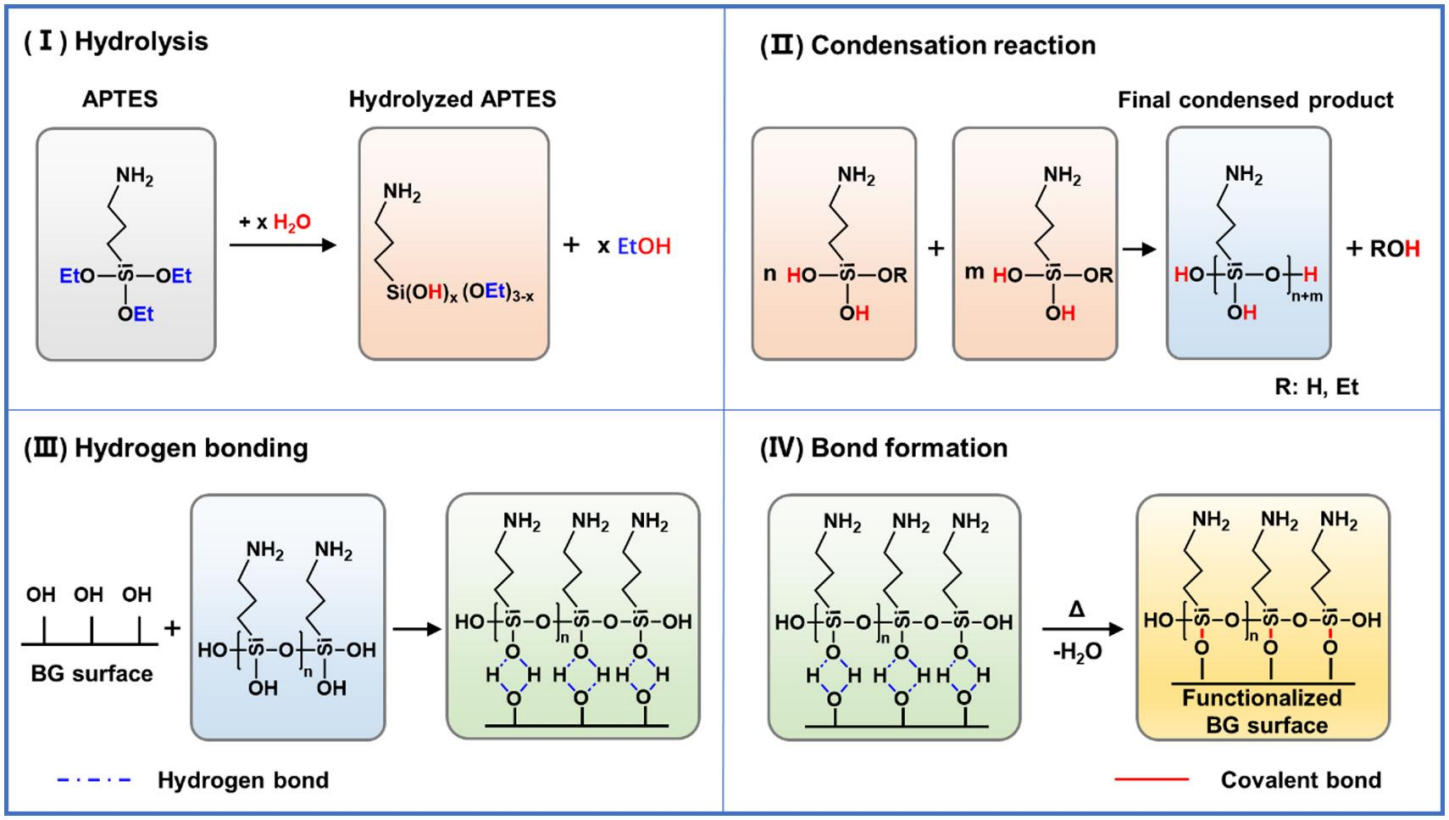
Surface modification of bioactive glass with APTES divided into (I) hydrolysis, (II) condensation reaction, (III) hydrogen bonding and (IV) bond formation [71] Copyright 2019, MDPI.

Among various substances used for surface modification of BGN, the silane coupling agent has become one of the most widely used reagents [69]. The different covalent coupling strategies are shown in Table 1. As early as 2006, Professor Yingjun Wang proposed to use 3-aminopropyltriethoxysilane (APTES or KH550) to treat the surface of BGN, and successfully introduced the coupling agent to the surface of BGN through the Si-O-Si bond (recorded as ABGN) [70]. The surface functionalization process of BGN by APTES can be divided into four steps: hydrolysis, condensation reaction, hydrogen bonding, and bond formation, as shown in Figure 3. In the water environment, APTES hydrolyzes to generate Si-OH groups, which can self-condense to form a Si-O-Si network. The exposed Si-OH groups combine with the Si-OH on the BGN surface through hydrogen bonding and undergo a condensation reaction, thus connecting APTES to the BGN surface to obtain ABGN [71]. The surface functionalization process using a silane coupling agent is simple, direct and inexpensive. The most important thing is introducing the active amino group (-NH_2_) to the BGN surface by covalent coupling. On the one hand, it can enhance the interface compatibility between BGN and the organic phase. On the other hand, -NH_2_ can be used as the active site for further grafting to introduce other multifunctional molecules to the BGN surface.

**Table 1.** Covalent coupling method for BGN modification

Type of coupling agent	Other components	The functions and applications	Ref.
3-aminopropyltriethoxysilane (APTES or KH550)	Glutaraldehyde (GA)	GA forms an imine bond with the -NH_2_ group on the surface of nanoparticles.	Gruian [72]
APTES	Aldehyde-based alginic acid	Excellent rheological properties, improved shape fidelity, and structural stability.	Zhu [73]
APTES	Collagen	The extension of cytoskeleton was enhanced in bone tissue engineering.	El-Fiqi [74]
APTES	Peptide (PR1P) and growth factor VEGF_165_	Overcome the problem of insufficient neovascularization in the regeneration of large bone defects.	Schumacher [75]
APTES	GelMA	A promising strategy for developing advanced periosteal biomaterials with good hand feel and bone repair performance.	Xin [76]
APTES	Folic acid (FA) and methotrexate (MTX)	It can effectively target the surface folic acid receptor of tumor cells to achieve tumor-killing effect.	Chen [77]
APTES	Maleic anhydride	Introduce -COOH active group on the surface of ABGN.	Aina [78]
APTES	Bromine-containing AIEgens	Strong blue fluorescence, ordered mesoporous structure and high *in vitro* biological activity.	Li [79]
			Wang [80]
APTES	Ag	Effective bacteriostasis against *Escherichia coli* and *Staphylococcus aureus.*	Zhu [81]
Vinyl trimethoxysilane (YDH171)	PLGA and poly(cyclopropane carbonate) (PTMC)	Improved the mechanical properties and crystallinity of the composite.	Qi [82]
Sodium carboxyethyl silane triol	Mono-(6-amino-6-deoxy)-β-Cyclodextrin（β-CD-NH_2_)	Increase the affinity for β-estradiol drugs in bone tissue regeneration.	Wang [83]
3-epoxypropylpropyltrimethoxysilane (GPTMS)		Enhances its antibacterial ability and fluorescence characteristics.	Akbari [84]
Lactide		It is conducive to the growth and proliferation of osteoblasts on the material surface and improves the biological activity of polyester materials.	Dong [85]
Sulfhydryl (SH)	Au	AuBGN is coated by cysteamine through selective sulfhydryl chemical adsorption.	Aina [86, 87]

**Figure 4.**
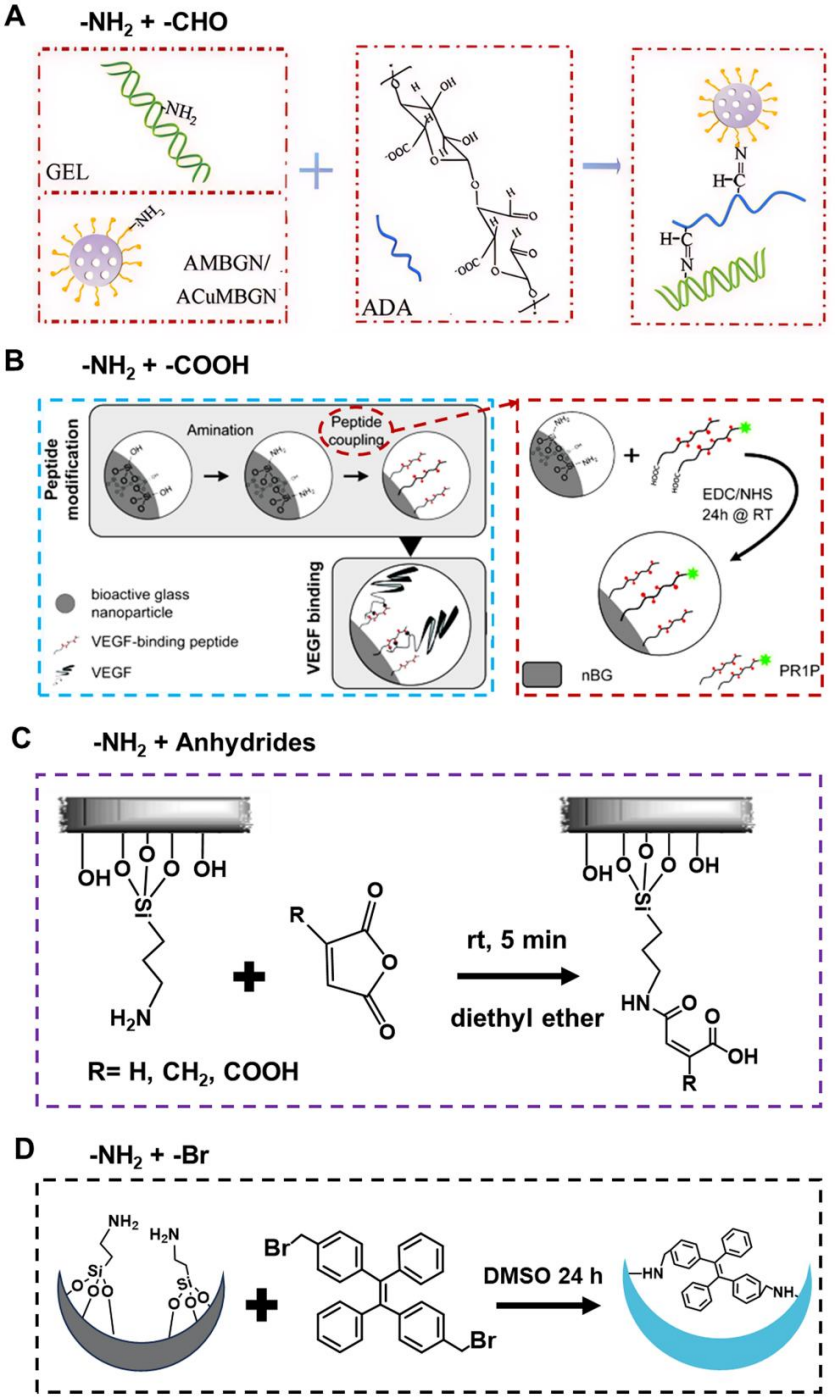
Reaction of aminated BGN (ABGN) with aldehyde group (**A**) [73] Copyright 2022, Wiley; carboxyl group (**B**) [75] Copyright 2022, American Chemical Society; anhydride (**C**) [78] Copyright 2014, American Chemical Society; and halogen (**D**) [80] Copyright 2016, Royal Society of Chemistry.

Based on the modification of APTES, ABGN was further functionalized by the chemical reaction of -NH_2_ on the surface of ABGN with other active groups to obtain BGN with multi-function. The -NH_2_ on the surface of ABGN can react with molecules containing aldehyde group (-CHO), carboxyl group (-COOH), anhydride and halogen or coordinate with metal ions to introduce active molecules to the surface of ABGN (Figure 4). For example, glutaraldehyde (GA) is anchored on the surface of ABGN through APTES as an intermediate. In this process, GA forms an imine bond with the -NH_2_ group on the surface of nanoparticles. However, it should be noted that if GA does not bind to the sample surface, it will show certain cytotoxicity [72]. Zhu *et al.* developed a multifunctional nanocomposite biological ink through 3D printing technology based on the Schiff base reaction between the amino groups on the ABGN surface and the aldehyde-based alginic acid. The hydrogel relies on dynamic covalent chemical bonds, and has excellent rheological properties, improved shape fidelity, and structural stability (Figure 4A) [73]. In the research of Zhu *et al.*, there is a close relationship between the surface modification of BGN and the preparation and performance of hydrogel. Firstly, the surface of BGN is modified to introduce specific functional groups, - NH_2_. This surface modification is based on the original chemical structure and properties of BGN, and the required functional groups are grafted onto its surface through chemical reactions such as silanization. The introduction of amino groups provides active sites for subsequent chemical reactions, allowing BGN to undergo Schiff base reactions with aldehyde functionalized alginate, forming dynamic covalent bonds (i.e. spontaneous Schiff base bonds). This dynamic covalent bond is stable and has a certain degree of reversibility, which is particularly important for preparing materials with adaptability and responsiveness. In this study, the amino groups on the surface of BGN tightly combined with the aldehyde groups on alginate through Schiff base reaction, forming a stable nanocomposite structure. Zhu *et al.* prepared multifunctional nanocomposite bio-ink through 3D printing technology based on the above-mentioned nanocomposite structure. Through surface modification and Schiff base reaction, Zhu and others successfully combined the excellent properties of BGN (such as biocompatibility, degradability, ion release ability) with the natural properties of alginate (such as good adhesion, moisture retention) to prepare a hydrogel preparation with multiple functions. This hydrogel has good mechanical properties and stability and can play a specific therapeutic role *in vivo*, promoting cell proliferation, differentiation, and tissue repair. The surface modification of BGN provides the basis for the preparation of hydrogel, and the Schiff base reaction is used as a bridge to connect BGN and alginate, realizing the close combination and performance optimization between the two. The development of this nanocomposite bioink provides new possibilities and application prospects for 3D printing technology in the biomedical field. El-Fiqi *et al.* developed a new nanocomposite hydrogel (Col–mBGN) made of collagen and mesoporous bioactive glass nanoparticles (mBGN) with surface amination. The amination of mBGN was proved to be able to chemically combine with collagen molecules, and the hydrolysis and enzymatic degradation of Col–mBGN hydrogel slowed down, and the extension of cytoskeleton was enhanced, which was conducive to the application of stem cell culture in bone tissue engineering [74]. In addition, Schumacher and others also introduced the binding peptide (PR1P) to the BGN surface based on the carboxyl reaction of amino and polypeptide in ABGN. They further fixed the angiogenic growth factor VEGF_165_ on the BGN surface in its natural state. The described nano system uses binding peptides to fix growth factors on BGN for the first time. It represents a promising strategy to overcome the problem of insufficient neovascularization in the regeneration of large bone defects (Figure 4B) [75]. Recently, Xin *et al.* constructed an inorganic/organic co-crosslinked double network hydrogel for bone defect repair by using the reaction of the amino group on the surface of ABGN and the carboxyl group on the GelMA chain, providing a promising strategy for developing advanced periosteal biomaterials with good hand feel and bone repair performance [76]. In addition, folic acid (FA) and methotrexate (MTX) are targeted molecules and anticancer drugs respectively, which are covalently coupled to the surface of ABGN through an amidation reaction, so as to prepare a nanosystem with targeted drug delivery (MTX-BGN-FA), which can effectively target the surface folic acid receptor of tumor cells to achieve tumor-killing effect [77]. In addition, maleic anhydride is also one of the commonly used materials for surface modification of inorganic materials. Maleic anhydride double bond opens and reacts with ABGN, so as to introduce -COOH active group on the surface of ABGN, facilitating further grafting of biological protein molecules containing sulfhydryl or amino groups (Figure 4C) [78].

In addition, the substitution reaction of halogen and an amino group can also realize the further modification of ABGN. Li *et al.* prepared ABGN and grafted bromine-containing aggregation-induced luminescence molecules (AIEgens, BTPE) onto ABGN through a substitution reaction. The prepared hybrid materials have strong blue fluorescence, ordered mesoporous structure and high *in vitro* biological activity, and can be used as drug carriers to deliver doxorubicin hydrochloride (DOX) in a slightly acidic environment (Figure 4D) [79, 80]. In addition, Zhu *et al.* aminated BGN with APTES and then soaked it in the solution containing Ag^+^ ions. Due to the coordination between amino and Ag^+^ ions, the loading of Ag^+^ was increased, and effective bacteriostasis against *Escherichia coli* and *Staphylococcus aureus* was achieved [81].

In addition to the APTES coupling agent, other siloxane coupling agents are also used to modify BGN. These include vinyl trimethoxysilane (YDH171), sodium carboxyethyl silane triol and 3-epoxypropylpropyltrimethoxysilane (GPTMS). The addition of YDH171 modified BGN (YDH-BGN) to PLGA and poly (cyclopropane carbonate) (PTMC) supports improved the mechanical properties and crystallinity of the composite. With the addition of YDH-BGN, the contact angle of the ternary composite increased [82]. In addition, the co-condensation reaction of sodium carboxyethyl silane trioxide and TEOS was used to introduce carboxylic acid to the surface of BGN, further utilizing mono-(6-amino-6-deoxy)-β-Cyclodextrin (β-CD-NH_2_) is functionalized to increase the affinity for β-estradiol drugs, and finally achieve the role of local administration for osteoporosis patients and promote bone tissue regeneration [83]. The modification of GPTMS on the surface of BGN not only enhances its antibacterial ability but also endows BGN with fluorescence characteristics [84].

In addition to silane coupling agents for covalent coupling, organic polymers can also be used to modify the surface of BGN. Due to the presence of Si-OH on the surface of BGN, lactide and hydroxyl can undergo ring-opening polymerization under the action of the catalyst, thus forming polylactic acid molecules on the surface of BGN. Composites of the modified PLLA-g-BGN with PLGA can improve the surface interface properties of polyester materials, as they are conducive to the growth and proliferation of osteoblasts on the material surface and improve the biological activity of polyester materials [85]. In addition, the covalent action of Au and sulfhydryl (SH) is also a method to modify BGN. First, the BGN containing Au (AuBGN) is prepared, and then the AuBGN is coated by cysteamine through selective sulfhydryl chemical adsorption, so that the amino group in the cysteamine molecule is exposed on the surface of AuBGN, realizing the amination modification of AuBGN [86, 87]. The advantage of the covalent coupling method is that the modified body can be tightly bound with BGN and is not easy to fall off, but is limited by the content of Si-OH on the surface.

### Metal coordination coupling

**Figure 5.**
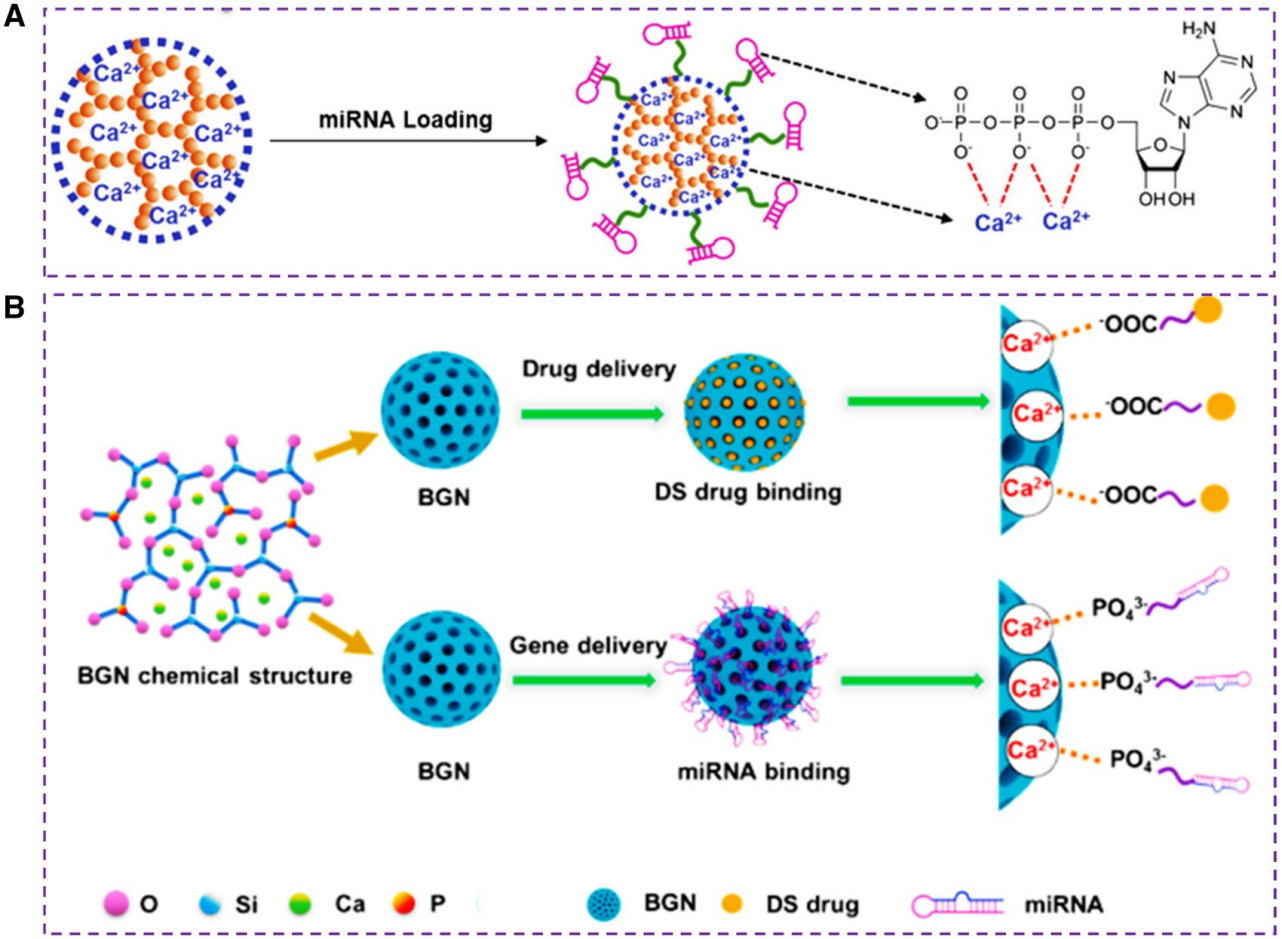
Ion interaction between RNA/drugs and BGN. (**A**) BGN loaded miRNA [95] Copyright 2017, Wiley. (**B**) BGN loaded diclofenac sodium (DS) drug and miRNA [96] Copyright 2014, American Chemical Society.

Because the structure of BGN contains metal cations, it is hopeful to modify the surface of BGN by metal coordination coupling. Table 2 shows different ion binding modes. Bioactive glass microspheres were prepared by crosslinking alginate with Ca^2+^ in BGN. After immersion in SBF, apatite was found on the surface of BGN alginate microspheres. As a drug carrier, it shows a sustainable drug release behavior and becomes a promising candidate material for the treatment of bone cancer [88]. Secondly, lecithin is a biosurfactant with a phosphate group as a hydrophilic group, with an amphiphilic molecular structure and interfacial adsorption capacity. The hydrophilic head can bond with the surface of BGN by an ionic bond. At the same time, the hydrophobic non-polar tail has excellent compatibility with chitosan molecules, which promotes the complete infiltration of the surface of the matrix and inorganic particles. It can significantly improve the interface bonding performance between bioglass and chitosan [89]. In addition, stearic acid is a high-grade fatty acid extracted from animal and vegetable oils. It is non-toxic and widely used for surface modification of inorganic materials. The carboxyl group at one end of its molecule can form a chemical bond with inorganic materials, and the long-chain alkyl structure at the other end is similar to the polymer molecular structure, making it have superior compatibility with polymer materials. The surface energy of BGN is effectively reduced by the local coating of the functional groups of organic molecules on the surface of the powder so that the powder is evenly dispersed and the agglomeration phenomenon is significantly improved. Therefore, the surface modification of stearic acid improves the surface properties and aggregation state of BGN, and this improvement in dispersion will also be more beneficial to the properties of the composite. In the porous scaffold of bioglass/chitosan composite, the dispersion of the modified BGN in the organic matrix phase was also significantly improved [90]. In addition, Bonici *et al.* utilized the interaction between thiol groups and Cu^2+^ ions to functionalize the surface of BGN by organic molecules (thiols) [91]. Additionally, the phosphate radical in the molecular structure of β-sodium glycerophosphate (GP) and sodium alendronate (AL) can combine with Ca^2+^ in BGN. GP was first introduced as a functional ligand to improve the stability and osteogenic differentiation of adipose stem cells and bone regeneration *in vivo* of BGN by Xue and Guo *et al.* [66, 92]. On this basis, Niu *et al.* combined folic acid with AL (FA-AL) and then modified the surface of BGN with it, endowing BGN with a targeted function [61]. In addition, Diba *et al.* immersed 45S5 BGN particles in AL solutions to form AL-cation coordination complexes and the hybrid particles facilitated the controlled release of anti-osteoporotic AL molecules and BG-derived inorganic ions, and displayed a strong anti-osteoclastic effect *in vitro*. *In vivo* evaluation of the hybrid particles revealed their strong capacity to facilitate the regeneration of bone defects in an osteoporotic rat model [93]. Boanini *et al.* have developed a mesoporous BGN with a loading capacity of up to 17 wt% of alendronate sodium. The results of *in vitro* experiments have shown that this multifunctional system is a promising platform for regulating the activity of osteosarcoma cells and osteoclasts, providing a new tool for treating osteosarcoma [94]. Similarly, miRNA and diclofenac sodium drugs can also be effectively loaded by ionic bond interaction due to the phosphate radical in miRNA and carboxylate group in diclofenac sodium drugs, which improves miRNA and drugs loading capacity compared to traditional silica nanoparticles (Figure 5) [95, 96]. This metal coordination coupling method is simple and easy to obtain. However, it will sacrifice the drug-carrying capacity of BGN, because when other materials modify the surface of BGN, its surface mesopores will be covered, limiting small molecule drugs to enter the porous interior.

**Table 2.** Metal coordination coupling method for BGN modification

Type of coupling agent	Metal cations	The functions and applications	Ref.
Alginate	Ca^2+^	It shows a sustainable drug release behavior.	Zhang [88]
Lecithin	Ca^2+^	Promotes the complete infiltration of the surface of the matrix and inorganic particles.	Zhao [89]
Stearic acid	Ca^2+^	The surface energy of BGN is effectively reduced and the powder is evenly dispersed.	Guo [90]
Sulfhydryl (SH)	Cu^2+^	The functionalized sol-gel Cu^2+^-containing glasses and are still Bioactive.	Bonici [91]
β-glycerol phosphate disodium salt (GP)	Ca^2+^	Improve the stability and osteogenic differentiation of adipose stem cells.	Xue [66], Guo [92]
Sodium alendronate (AL)	Ca^2+^	Strong capacity to facilitate regeneration of bone defects	Diba [93]
Sodium alendronate (AL)	Ca^2+^	Regulating the activity of osteosarcoma cells and osteoclasts	Boanini [94]
miRNA	Ca^2+^	Increase the load of miRNA	Xue [95]
Diclofenac sodium	Ca^2+^	Increase the load of diclofenac sodium drug	Yu [96]

### Physical adsorption

BGN has a large specific surface area due to its inherent nano-size effect and surface mesoporous structure, which enables BGN to achieve surface modification of BGN through physical adsorption. The adsorption is mainly realized by the hydrophilic hydrophobic interaction, electrostatic interaction and hydrogen bonding. Typically, this method attaches biological molecules such as lipids, proteins, antibodies, or polymers. Non-covalent linking is a simple and inexpensive method that can conduct surface exchange reactions to replace the previously linked molecules with other molecules with high affinity to the BGN surface, but no special affinity to the cells under study. Chen *et al.* prepared BGN polyelectrolyte composite (composed of carboxymethyl starch and chitosan oligosaccharide) nanocomposites (BGN/PEC) with different BGN contents by *in situ* coprecipitation method and freeze-drying method. With the addition of BGN, its degradation *in vitro* was significantly improved, and a more neutral environment was obtained, which was more suitable for surgical application [97]. In addition, Aguilar *et al*. combined BGN and polyhydroxyalkanoate microspheres for drug loading. The composite microspheres have a curcumin loading efficiency of more than 90%, biological activity and curcumin release capacity, achieving potential application prospects in the biomedical field [98]. Richard *et al.* used the high specific surface area of BGN as the carrier system of lysozyme or growth factor, successfully loaded lysozyme onto the surface of BGN through mixing and stirring, further constructed calcium phosphate bone cement and lysozyme functionalized BGN composite, making it as a promising candidate for bone tissue engineering [99]. Additionally, Chen *et al.* modified the FBS protein onto the surface of BGN, achieving outstanding dispersion stability of BGN particles [53]. BGN is surface modified by the physical adsorption effect. The BGN modified by this method is unstable, and the surface substance will fall off within a certain period, which is not conducive to the long-term use of BGN. In addition, for the adsorption of proteins, it is not ruled out that the carboxylate groups in the protein structure may interact with the ions in BGN.

While exploring the interaction between BGN and proteins, we have learned that multiple vital factors affect the adsorption behavior of proteins, including material composition, structural properties, surface charge, hydrophilicity, crystallinity and the characteristics of the protein itself. These interactions not only determine the biological reactivity and solubility of biomaterials but also directly affect the activity and behavior of cells on the material surface, such as biomineralization and cell adhesion [100, 101].

Composition and structure of materials: Introducing elements such as Ca into silicate structures can reduce network connectivity, improve the surface activity and dissolution rate of bioactive glass, and promote the formation of mineralized products such as hyaluronic acid, which can more effectively bind to protein molecules [102, 103]. The distribution of porosity and pore size also significantly affects the adsorption process, with larger pore sizes facilitating the adsorption of high molecular weight proteins [104, 105]. In addition, when the size of nanoparticles is finely reduced, their specific surface area (SSA) increases sharply, providing more abundant binding interfaces for biomolecules such as proteins, thereby significantly increasing the amount and rate of protein adsorption on the surface of nanoparticles [106]. Of particular concern is that as the particle size further shrinks below a specific threshold (usually less than 100 nanometers), a more complex and important phenomenon begins to emerge-the formation of protein coronations [107]. However, current research on the specific mechanisms and effects of protein corona formation still needs to be completed. The research gap in this field limits our comprehensive understanding of the interactions between nanoparticles and biological interfaces, and also hinders the further development and optimization of nanoparticle-based biomaterials and technologies in clinical applications.

*Surface charge*: BGN usually carries a negative charge in physiological media, while the surface charge of proteins varies due to their asymmetric charge distribution within the molecule. This charge difference may lead to electrostatic repulsion or attraction, thereby affecting the adsorption quantity and structure of proteins. However, the positive and negative charge sites on the surface of BGN (such as Ca^2+^ and OH^−^) may also form electrostatic bonds with charged functional groups in protein molecules, enhancing or inhibiting adsorption [108, 109].

*Hydrophilicity and hydrophobicity*: Protein is more inclined to adsorb on hydrophobic surfaces, but serum proteins (such as vitreous adhesion proteins) are more likely to adsorb on hydrophilic surfaces [110, 111]. Therefore, the hydrophilic hydrophobic balance of BG has a significant impact on protein adsorption.

*Crystallinity*: BGN with high crystallinity may decrease their affinity for negatively charged proteins due to increased surface negativity, and may cause conformational changes in adsorbed proteins, inhibiting cell adhesion. In contrast, the amorphous BGN surface can adsorb more serum proteins, which is beneficial for cell adhesion [112, 113].

*Protein interaction*: During the process of adsorption onto the BGN surface, proteins not only interact with the material surface but also with other protein molecules [109]. This interaction can generate cooperative or competitive effects, affecting the adsorption of specific proteins on the surface. The positive synergistic adsorption indicates that adsorbed proteins can ‘attract’ more similar proteins to the surface, which provides the possibility of enhancing specific protein adsorption [114]. However, the mechanism of this synergistic effect still needs to be fully understood, and further research is needed to develop more effective surface modification strategies.

*The impact of surface modification*: Surface modification of BGN using proteins is a promising method that can reduce protein activity loss and improve biocompatibility. By selectively adsorbing or binding proteins with specific functions (such as growth factors, adhesion proteins), the behavior of cells on material surfaces can be regulated, promoting tissue regeneration and repair. In addition, surface modification can improve the targeting and stability of BGN and prolong its *in vivo* action time [53, 66].

In summary, the interaction between BGN and proteins is a complex and multifactorial process involving the physical and chemical properties of materials, the characteristics of proteins, and their interactions. By deeply understanding and optimizing these interactions, biomaterials with better biocompatibility and functionality can be developed, bringing innovation and development to the biomedical field. Future research should further explore the molecular mechanisms, synergistic effects, and surface modification strategies of protein adsorption to achieve more precise material design and applications. Additionally, specific details regarding the interaction between proteins and bioactive glass surfaces can be found in the relevant literature by Aldo R. Boccaccini’s team.

### *In situ* surface polymerization

**Figure 6.**
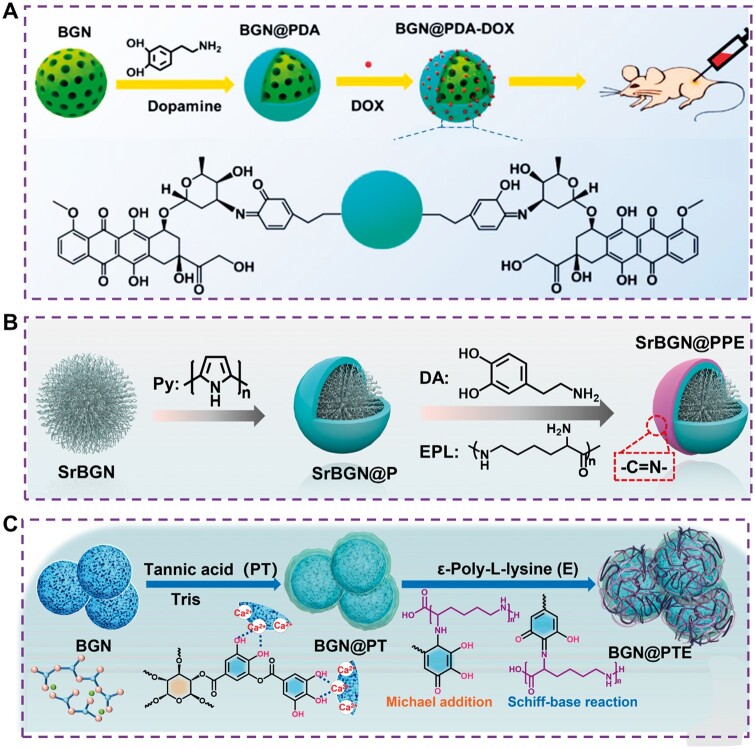
Modification of BGN by *in situ* polymerization. (**A**) Modification of BGN by dopamine (PDA) [68] Copyright 2020, American Chemical Society. (**B**) Modification of BGN by polypyrrole (Py), dopamine (DA) and ε-poly-l-lysine (EPL) [118] Copyright 2022, Elsevier. (**C**) Modification of BGN by tannic acid (PT) and ε-poly-l-lysine (E) [119] Copyright 2022, KeAi.

The essence of *in situ* polymerization is that soluble active monomer molecules gather *in situ* on the surface of nanoparticles and polymerize, thus forming a stable coating on the surface of nanoparticles. Polyphenols have become the most widely studied *in situ* polymerization-modified materials due to their remarkable biological functions (such as anti-oxidation, strengthening blood vessel walls, inhibiting bacteria and cancer cell growth) and easy oxidative polymerization. The rich functional groups (catechol, quinone, amine, imine) in the polydopamine coating (PDA) can change the surface properties and provide more functions for biological matrix materials. Xue *et al.* used PDA to assemble the surface of BGN to establish a stable near-infrared excited photothermal nano platform (BGN@PDA) for tumor ablation. BGN@PDA shows the loading capacity of high anticancer drugs (DOX) and the enhancement of tumor chemotherapy. At the same time, BGN@PDA can actively induce osteoblasts to form bone *in vitro* and has a strong ability to promote the repair of rat skull defects (Figure 6A) [68]. Similarly, Sarika Tomar used dopamine to modify BGN and further compounded it with curcumin nanoparticles/gelatin and chitosan to form a composite hydrogel, effectively promoting wound healing [115]. In addition, natural polyphenols and gallic acid extracted from red grape peels and green tea can also be used to modify the surface of BGN. It was found that functionalized BGN had good biological activity *in vitro*, and the grafting of tea polyphenols improved the antioxidant activity of BGN, which could be applied to tissue repair [116, 117]. In addition, after functionalizing BGN with seaweed polyphenols, silver nanoparticles (Ag NPs) are enriched on the surface of BGN through *in situ* reduction technology to obtain composite nanoparticles (BGN Ag NPs). The presence of polyphenols and Ag NPs significantly reduces the metabolic activity of *S.aureus* biofilm, and the BGN Ag NPs show good cytocompatibility with human osteoblast progenitor cells [116]. Chen *et al.* used polypyrrole and PDA to modify BGN *in situ* and then further used polydopamine to react with ε-poly-L-lysine (EPL) to modify EPL on the surface of BGN, thus realizing the multifunctional properties of BGN, such as antibacterial and antioxidant. It can be seen that natural polyphenol functionalized BGN is a promising biomaterial (Figure 6B) [118]. Additionally, in Wang’s work, a multilayer-structured bioactive glass nanosystem (BGN@PTE) is developed by coating the poly-tannic acid and ε-poly-L-lysine onto the BGN *via* facile layer-by-layer assembly as an integrative and multilevel dressing for the sequential management of wounds (Figure 6C) [119]. BGN was modified by *in situ* polymerization. The modified coating was stable and had a certain bionic function, but the degradation of BGN was slow after polymerization, which delayed its degradation.

## Multifunctional properties of BGN

**Figure 7.**
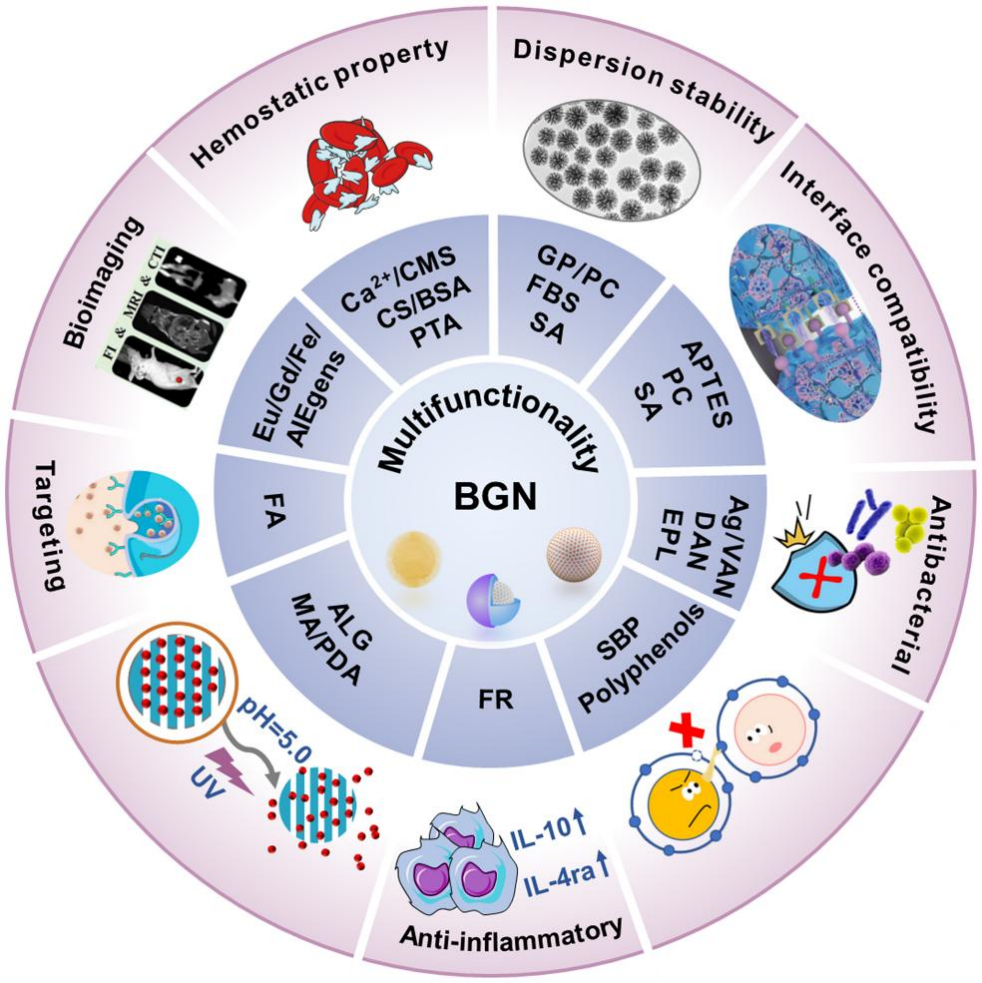
Multifunctionality of modified BGN.

In general, the surface modification of BGN, on the one hand, maintains the inherent characteristics of BGN (such as biological activity, degradability, etc.), on the other hand, can ‘customize’ new functional characteristics (such as antibacterial, antioxidant, anti-inflammatory, etc.) or improve the defects of BGN (such as easy agglomeration, poor compatibility with organic interfaces, *etc.*) to achieve a wider application of BGN. The performance of the modified BGN is shown in Figure 7.

### Dispersion stability

**Figure 8.**
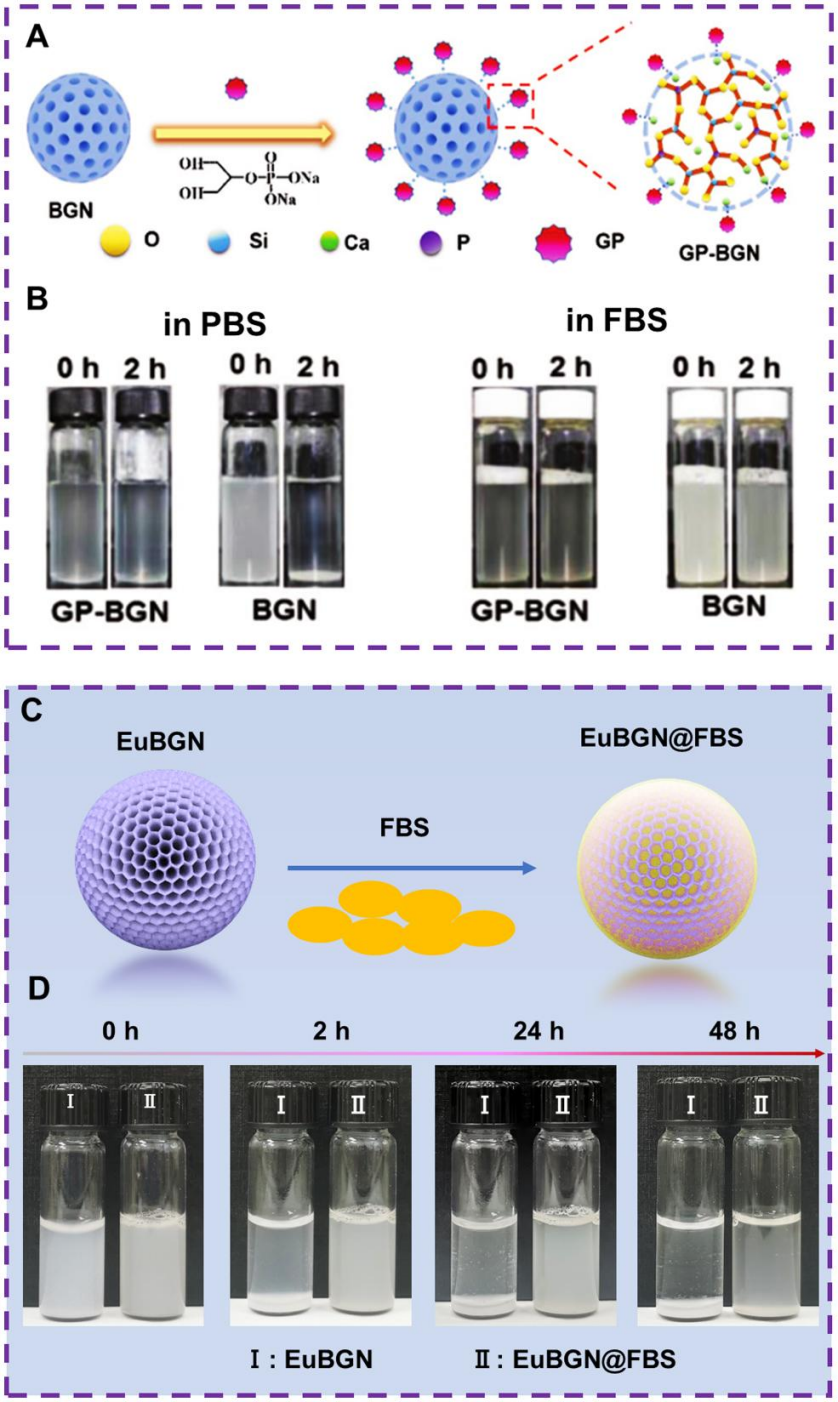
Dispersion stability of m-BGN. (**A**) Schematic diagram of β-sodium glycerophosphate (GP) modified BGN. (**B**) Dispersion stability of GP-BGN in PBS and FBS solutions [66] Copyright 2019, Wiley. (**C**) Schematic diagram of FBS modified EuBGN. (**D**) Dispersion stability of EuBGN@FBS in PBS solution [53] Copyright 2021, American Chemical Society.

Due to the advantages of nano biomaterials in terms of physical and chemical properties, monodisperse BGN has been developed as a potential drug/gene delivery carrier. However, BGN has poor physiological stability and is easy to reunite. In clinical application, it may cause non-specific binding, immune response and clearance by the mononuclear phagocytosis system, which severely limits the application of BGN in nano biomedicine. Therefore, it is imperative to improve the hydrophilicity and physiological stability of BGN and expand its application in disease imaging and cancer treatment. Xue *et al.* first introduced β-sodium glycerophosphate (GP) was used as a functional ligand to modify BGN to obtain GP-BGN, which improved the dispersion stability of BGN in PBS and FBS solutions (Figure 8A and B). In addition, the modification of GP also significantly enhanced the uptake efficiency of nanoparticles by cells, which provided an opportunity to improve drug utilization [66]. The introduction of GP may promote the uptake of BGN nanoparticles by cells through the following aspects: firstly, as a functional ligand, GP contains multiple hydrophilic groups (such as hydroxyl and phosphate groups) in its molecule, which can significantly enhance the surface hydrophilicity of BGN nanoparticles. The enhancement of hydrophilicity makes it easier for BGN nanoparticles to form a stable hydration layer with water molecules when interacting with cells, thereby reducing mutual attraction and aggregation between particles and promoting uniform distribution of particles around cells. Secondly, the hydrophilic surface makes it easier for BGN nanoparticles to interact with the phospholipid bilayer of the cell membrane, or attract charged groups on the cell membrane through their surface charge, thereby promoting the fusion or encapsulation of particles with the cell membrane. This process is called endocytosis, an important way for cells to take up foreign particles. Finally, the introduction of GP may also alter the surface chemical properties of BGN nanoparticles, making them more easily recognized and bound by receptors on the cell surface. Receptors on the surface of cells typically have specific binding sites that can recognize and bind to molecules or particles that match them. The BGN nanoparticles modified by GP may further promote the uptake of particles by cells through specific binding to cell surface receptors. In addition, surface modification with lecithin (PC) can effectively reduce the surface energy of BGN. The modified BGN has good dispersion and is not easy to agglomerate [89]. Guo *et al.* partially coated the surface of BGN with stearic acid (SA) to make BGN organic, effectively reducing the surface energy of BGN, making BGN disperse evenly, and significantly improving the agglomeration phenomenon [90]. Chen *et al.* reported that europium-doped BGN modified by fetal bovine serum (FBS) significantly enhanced the dispersion in physiological media, improved blood compatibility and cell uptake of BGN, and achieved targeted tumor imaging and treatment (Figure 8C and D) [53]. In summary, by introducing biosafety substances rich in hydrophilic groups, such as β-glycerophosphate, lecithin, stearic acid, and fetal bovine serum, researchers have successfully improved the monodispersity stability and physiological properties of BGN, laying a solid foundation for its application in biomedical fields such as disease imaging and cancer treatment.

Researchers have employed various techniques to evaluate the long-term stability and biosafety of modified BGNs, such as GP-BGN and FBS-BGN. Xue *et al.* systematically monitored the stability of GP-BGN *in vitro* and *in vivo* using TEM (transmission electron microscopy) and ICP (inductively coupled plasma mass spectrometry) techniques. They not only observed changes in particle morphology, but also quantitatively analyzed ion release, and detected the content of Si ions and histological changes in different organs through *in vivo* experiments in mice, providing strong evidence for the long-term stability and biosafety of GP-BGN [66]. Chen *et al.* employed TEM, FTIR (Fourier transform infrared spectroscopy) and SDS-PAGE (sodium dodecyl sulfate polyacrylamide gel electrophoresis) to assess the structural stability of FBS-BGN. However, their research on the long-term stability *in vivo* remains insufficient. This underscores the importance of considering a combination of *in vivo* and *in vitro* experiments when evaluating the long-term effects of modified nanomaterials. This comprehensive approach is crucial for a thorough assessment of their safety and effectiveness [53].

In addition, there may be other innovative methods to more intuitively track the distribution and long-term stability of BGN within cells. We speculate that fluorescent dye labeling technology can monitor the dynamic changes of BGN in cells in real time through fluorescence microscopy or flow cytometry. However, fluorescent dyes may have certain cytotoxicity and, in some cases, may interfere with the normal physiological functions of cells. Radioactive isotope labeling technology provides a more accurate method for studies requiring higher sensitivity and specificity. By measuring the radioactive activity in the sample, precise tracking of the fate of BGN in cells can be achieved. However, this method requires high experimental conditions and technical requirements, and there are radiation safety hazards that require careful operation. In addition, mass spectrometry analysis technology is also a powerful tool for evaluating the degradation or metabolism of BGN in cells. By detecting the types and quantities of degradation or metabolic products, the long-term stability of BGN can be indirectly inferred. This method has high specificity and sensitivity, but also requires complex sample processing and analysis processes.

In summary, evaluating the long-term stability and biosafety of modified BGN requires the comprehensive use of multiple technical methods combined with *in vivo* and *in vitro* experimental results for comprehensive analysis. Future research can further explore more innovative methods to improve the accuracy and reliability of evaluations.

### Interface compatibility

BGN is usually used as the filling material of various organic scaffolds. However, the difference between the BGN inorganic phase and the polymer organic phase in terms of hydrophilicity and hydrophobicity makes the two phases lack a suitable bonding interface, resulting in low bonding strength between the two phases and even BGN particle stripping, which affects the strength of composite scaffolds. Therefore, surface modification of BGN to improve its interfacial compatibility is particularly important. The surface amination of BGN became the main strategy. Studies have shown that the mechanical properties of the composite scaffold obtained by using APTES to modify the surface of BGN and then compounding aminated BGN with chitosan gelatin are higher than those of the unmodified composite scaffold, and it is observed by the microscope that the composite porous scaffold has strong two-phase compatibility and tight interface bonding; The pores of the scaffold are connected and arranged regularly, which indicates that the affinity between BGN and polymer phase has been improved, further indicating that amination can improve the interface compatibility of composite scaffold [70]. A hyaluronic acid chitosan composite scaffold (BGN-HA/CS) was also prepared using the aminated BGN. The scaffold had an obvious porous structure, and the pores are highly connected, which is conducive to the transport of nutrients and drugs required by cells [120]. In addition, the dispersion of BGN is improved, and the interface compatibility of BGN in the organic phase is also improved. For example, using lecithin or stearic acid to modify BGN can make the dispersion of BGN uniform and attenuate the aggregation of BGN. Further, the modified BGN was added to the chitosan scaffold, and BGN and chitosan could fuse well, indicating good interfacial compatibility between BGN and chitosan [89, 90].

**Figure 9.**
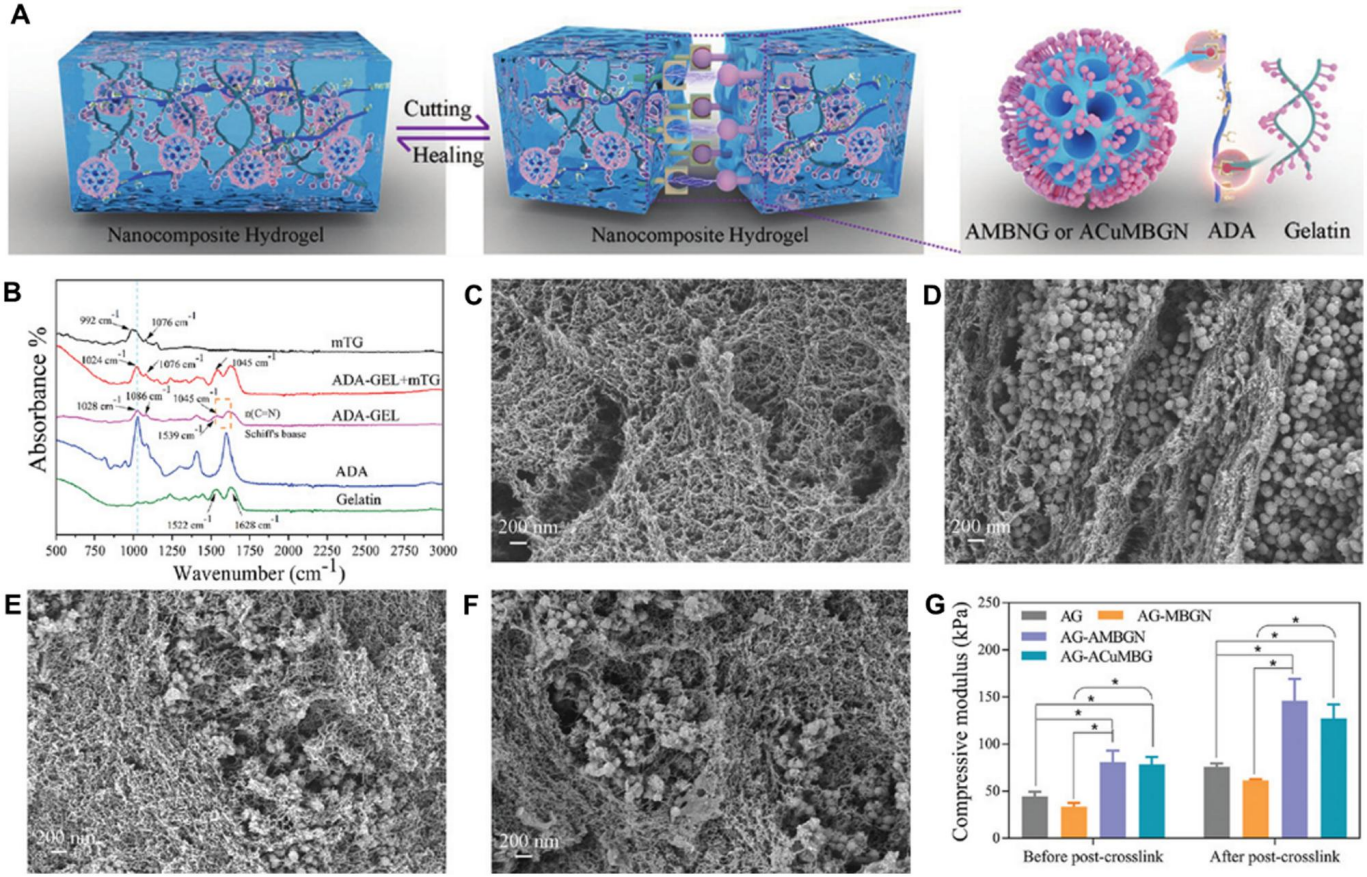
Interface interaction between BGN and hydrogel matrix. (**A**) Proposed mechanism of self-healing in AG-AMBGN or ACuMBGN hydrogels. (**B**) FTIR analysis of ADA-GEL hydrogels before and after post-crosslinking. SEM images of (**C**) AG, (**D**) AG-MBGN, (**E**) AG-AMBGN, and (**F**) AG-ACuMBGN samples after post-crosslinking. (**G**) The compressive modulus of the four groups before and after post-crosslinking, *n* = 3, *P < 0.05 [73] Copyright 2022, Wiley.

In addition, when evaluating the interfacial compatibility between BGN and polymer matrix, multiple testing methods must be used to understand the interfacial bonding situation comprehensively. Various methods, such as high-resolution microscope observation, mechanical property testing, and chemical characterization, can be considered to evaluate interface compatibility comprehensively. These methods can reveal the details and characteristics of interface bonding from different perspectives, providing strong support for optimizing the composite process of BGN and polymer matrix. Zhu *et al.* evaluated the binding performance of BGN with gelatin scaffolds using Fourier transform infrared spectroscopy (FTIR), scanning electron microscopy (SEM), and mechanical property testing. Figure 9A shows the self-healing mechanism diagram of BGN combining with aldehyde sodium alginate/gelatin to form a hydrogel. BGN realizes the dynamic self-healing function of hydrogel through the Schiff base reaction between the surface amino group and the aldehyde group in sodium alginate. Figure 9B–G shows the FTIR, SEM images and compression performance results of each sample, confirming that the aminated BGN can effectively combine with the hydrogel matrix through chemical bonds. Moreover, it is more evenly distributed and can improve the compression performance of composite scaffolds [73]. By aminating BGN and further reacting it with natural polymer scaffolds, the biphasic interface problem between inorganic nanoparticles and organic phases is improved, and the mechanical properties of the scaffolds are enhanced.

### Antibacterial property

**Figure 10.**
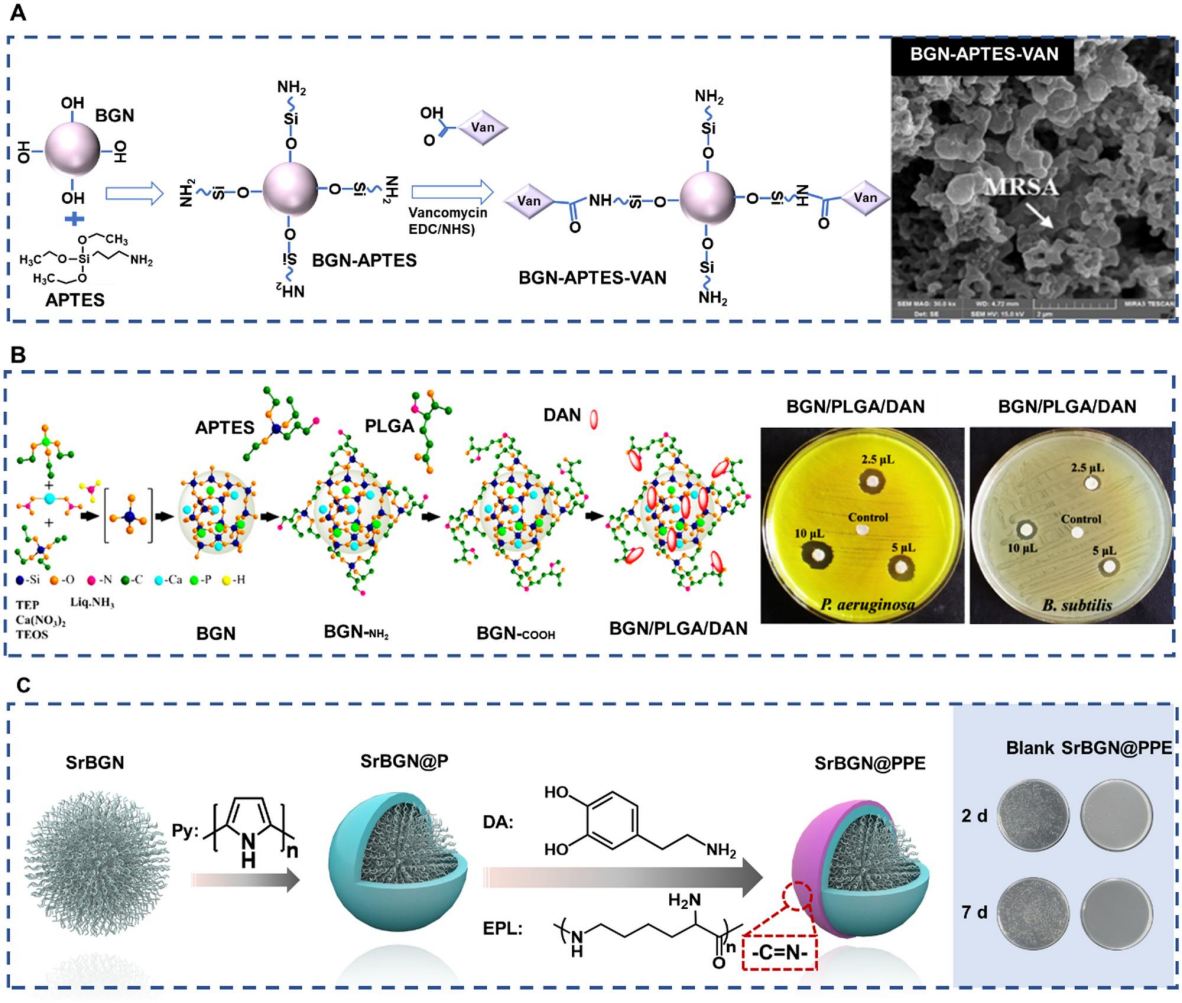
Antibacterial property of m-BGN. (**A**) Schematic diagram of vancomycin (VAN) modified BGN and antibacterial ability [121] Copyright 2020, Elsevier. (**B**) Schematic diagram of daunomycin (DAN) modified BGN and antibacterial ability [122] Copyright 2021, Elsevier. (**C**) Schematic diagram of ε-poly-L-lysine (EPL) modified BGN and antiinfection ability [118] Copyright 2022, Elsevier.

Postoperative bacterial infection is one of the major problems in clinical practice and even threatens the life safety of patients when it is serious. Therefore, we ‘customize’ an antibacterial coating for BGN, hoping to solve the infection problem in tissue repair. Ag has a significant antibacterial effect. The antibacterial effect of BGN can be effectively realized by enriching Ag on the surface of BGN through surface modification technology. After surface modification of BGN with APTES with an amino group, Ag^+^ is stabilized on the surface of BGN material through the coordination reaction of Ag^+^ and amino group, which improves the loading of Ag^+^ [81]. Secondly, based on the amination of BGN, vancomycin (VAN) was fixed on BGN through the EDC/NHS cross-linking process. BGN-APTES-VAN has strong bactericidal activity against methicillin-resistant *S.aureus* (MRSA) and has shown the ability to treat infection in the short term and long term [121] (Figure 10A). In the same way, polyglutamic acid (PLGA) was grafted onto the surface of BGN (BGN/PLGA), showing good dispersion and high drug loading effect, which can be used for loading of daunomycin (DAN, antibacterial drug), so that BGN/PLGA/DAN showed superior antibacterial activity against gram-negative bacteria [122] (Figure 10B). In addition, fluorination treatment of BGN can significantly improve its antibacterial activity. Nam *et al.* added NaF in the synthesis process to prepare FBGN, and then added FBGN to the clinical orthodontic resin, which can effectively prevent leukoplakia and inhibit the proliferation of *Streptococcus mutans* [123]. It is also a way to achieve antibacterial effect by introducing antibacterial agents onto the BGN surface through a chemical reaction. Chen *et al.* used Schiff base reaction to connect ε-poly-L-lysine (EPL) to polydopamine and polypyrrole-modified BGN, realizing the broad-spectrum antibacterial and anti-infective effects of BGN *in vivo* [118] (Figure 10C). The antibacterial mechanism of BGN (bioglass nanoparticles) after surface modification may involve multiple aspects such as enhanced ion release mechanism, promotion of bacterial cell membrane damage, induction of oxidative stress, enhancement of metabolic interference, and realization of synergistic antibacterial effect. However, the specific mechanism needs to be analyzed based on the different modification methods and materials used. In this article, the antibacterial properties of BGN were mainly discussed through surface modification methods, which involve using chemical reactions to graft antibacterial drugs or agents onto the surface of BGN, such as Ag ions, daunorubicin, NaF and polylysine. BGN modified in body fluids or water environments can release ions with antibacterial activity, which can penetrate bacterial cell membranes, disrupt cellular metabolic processes and functions, and lead to bacterial death. In addition, daunorubicin, NaF and polylysine on the surface of BGN may enhance their interaction with bacterial cell membranes by changing their surface properties (such as charge, hydrophilicity, hydrophobicity, etc.), thereby promoting damage to bacterial cell membranes.

### Antioxidant activity

**Figure 11.**
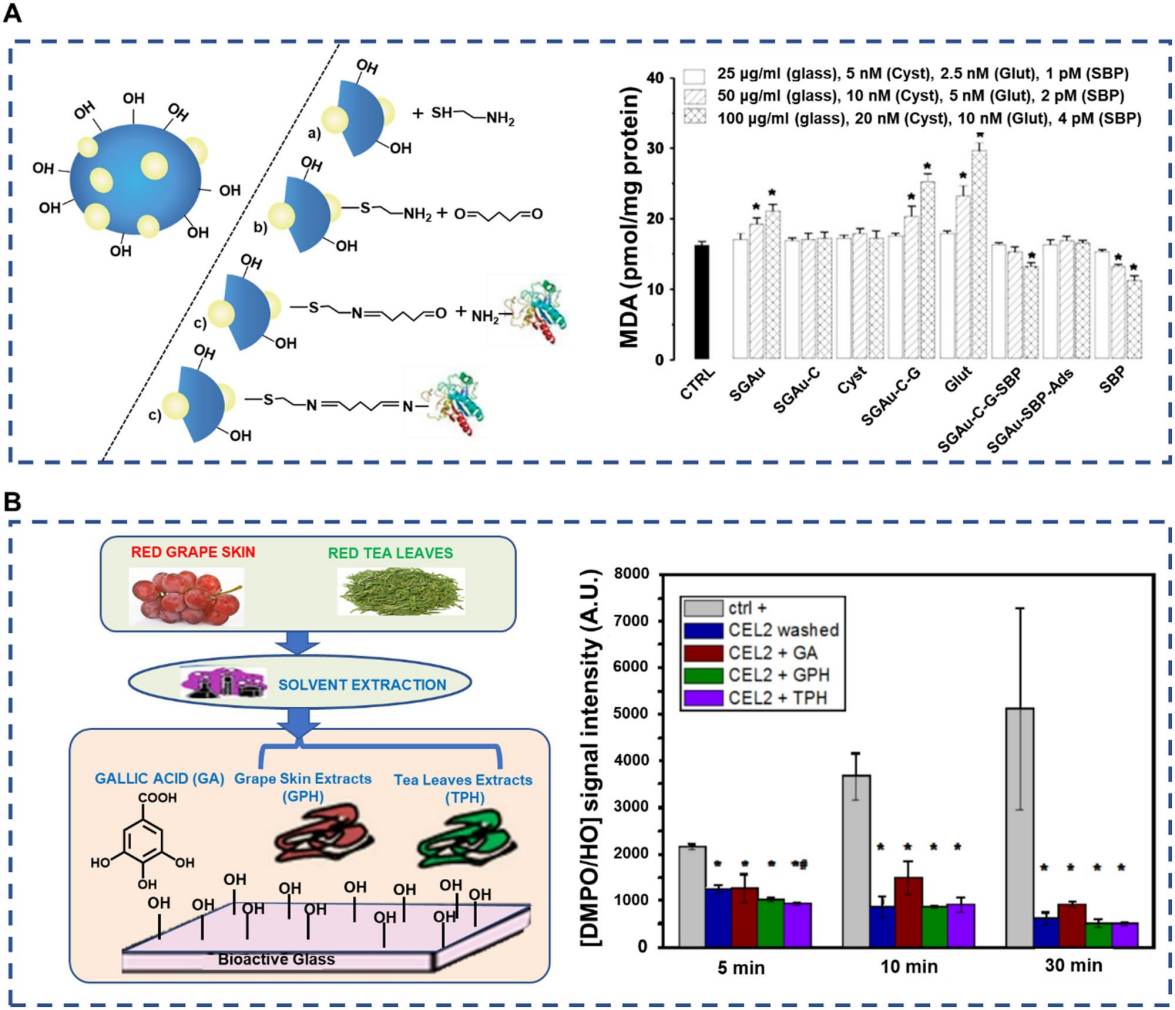
Antioxidant property of m-BGN. (**A**) Schematic diagram of soybean peroxidase (SBP) modified BGN and anti-oxidative stress ability [86] Copyright 2016, Royal Society of Chemistry. (**B**) Schematic diagram of polyphenol modified BGN and antioxidant ability [116] Copyright 2016, Elsevier.

The human body consumes oxygen for energy supply at all times and produces living oxygen free radicals (ROS), which play an important role in cell signal transduction [124]. Since ROS is an unstable substance lacking electrons, which will remove electrons from cell protein molecules, and excessive ROS will cause severe damage to cell structure, the antioxidant property of biomaterials is fundamental [125]. For example, Aina *et al.* built a new biological coupling material with antioxidant properties by layer-by-layer assembly. Different from other work that often uses the preliminary silylation process, they first introduced gold nanoparticles (AuNPs) into the BGN component, and then coated AuNPs with cysteamine through selective sulfhydryl chemical adsorption, thus introducing active amino groups to the BGN surface. Then, a continuous double coupling reaction was realized between the cysteine-modified AuNPs and the external amino groups available on soybean peroxidase (SBP) using a dialdehyde linker (glutaraldehyde), respectively. Thus, SBP was immobilized on the AuNPs containing bioglass through a covalent bond, and a new type of biological coupling material was obtained. This material can maintain its activity over time and reduce oxidative stress when human osteosarcoma cells (MG-63) contact [86] (Figure 11A). In addition, natural polyphenols have significant antioxidant properties, which can be effectively enhanced by introducing them to the surface of BGN through *in situ* polymerization [116] (Figure 11B). In summary, the improvement of BGN’s antioxidant function through surface modification is mainly achieved through covalent coupling by grafting antioxidant biomolecules onto the surface of BGN. This provides an effective solution to enhance the antioxidant performance of BGN and further broadens its application prospects in the biomedical field.

### Anti-inflammatory activity

**Figure 12.**
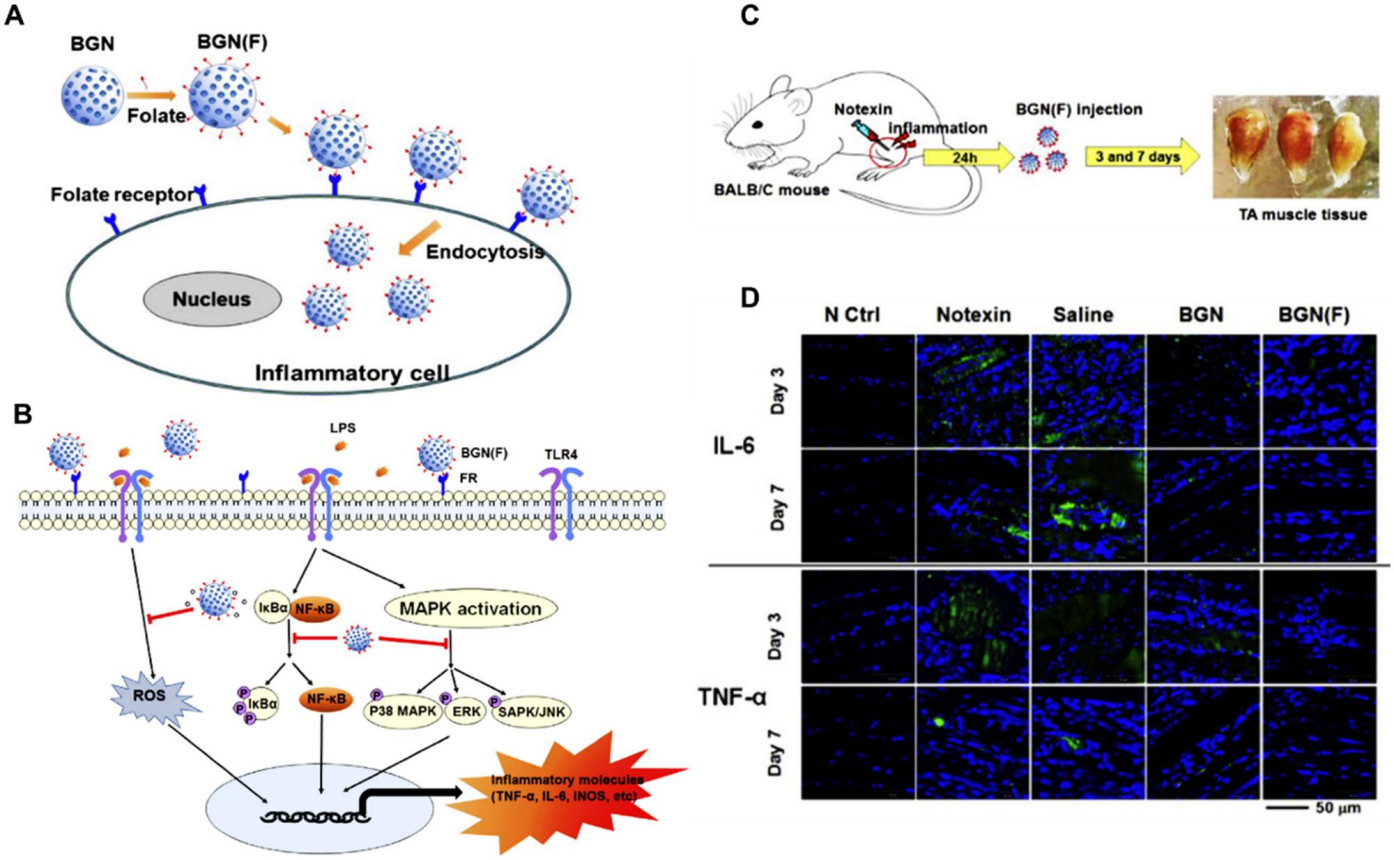
Anti-inflammatory activity of m-BGN. (**A**) Schematic illustration showing the interaction of BGN(F) with a targeted inflammatory activated cell. (**B**)Schematic illustration of the series of inflammatory-related signaling events in the LPS-activated macrophage upon interactions with the anti-inflammatory therapeutic nanoparticle BGN(F). (**C**) Schematic illustration of mouse tibialis anterior (TA) muscle injury model. (**D**) Immunohistochemical analysis of the IL-6 and TNF-α in tissue samples. Representative confocal microscopic images on days 3 and 7, revealing signals of IL-6 and TNF-α [132]. Copyright 2019, Elsevier.

Inflammation plays a vital role in the human body and is the most critical basic pathological process. When the inflammatory cytokines act on the body, the body eliminates the inflammatory factor through the inflammatory reaction. However, when the inflammatory reaction is out of control, it will harm human health. In particular, chronic inflammation, which is difficult to heal itself, may even lead to more severe diseases (such as cancer), seriously threatening human health [126]. The anti-inflammatory effect of BGN can further expand its application in inflammatory wounds. Studies have shown that folic acid (FA) has become a target ligand for cancer cells and inflammatory cells, because folic acid receptors (FRs) are overexpressed on the surface of activated cells, while the expression level in normal cells is low [127]. It is reported that when anti-inflammatory drugs are combined with folic acid, the targeting and anti-inflammatory activities are significantly improved [128–131]. Folic acid-functionalized BGN (FBGN) can combine with proinflammatory cells and release functional ions after endocytosis (Figure 12A). Folic acid coupling significantly enhanced the internalization of lipopolysaccharide (LPS)-induced proinflammatory cells by nanoparticles. Appropriate concentrations (80 and 160 μg ml^−1^) can save LPS-induced inflammatory cell activity, reduce cell death, prevent lactate dehydrogenase release and ROS production, and down-regulate proinflammatory factors (Figure 12B). In addition, FBGN can be administered locally to the muscle-injured tissues of mice induced by Notexin, thereby significantly down-regulation of IL-6 and TNF-α, conversion of macrophage phenotype from M1 to M2, and accelerating tissue healing (Figure 12C and D). FBGN is promising to be used as a new drug-free nanotherapeutic platform to alleviate inflammatory reactions [132]. Surface-modified BGN nanoparticles have shown great potential for anti-inflammatory effects. By implementing reasonable surface modification strategies, the targeting, biocompatibility, and functionality of BGN can be significantly enhanced, thus playing a more important role in the treatment of inflammatory diseases. In the future, with the deepening of research and continuous development of technology, surface-modified BGN nanoparticles are expected to achieve more significant results in the field of anti-inflammatory therapy.

### Controlled release of drugs

**Figure 13.**
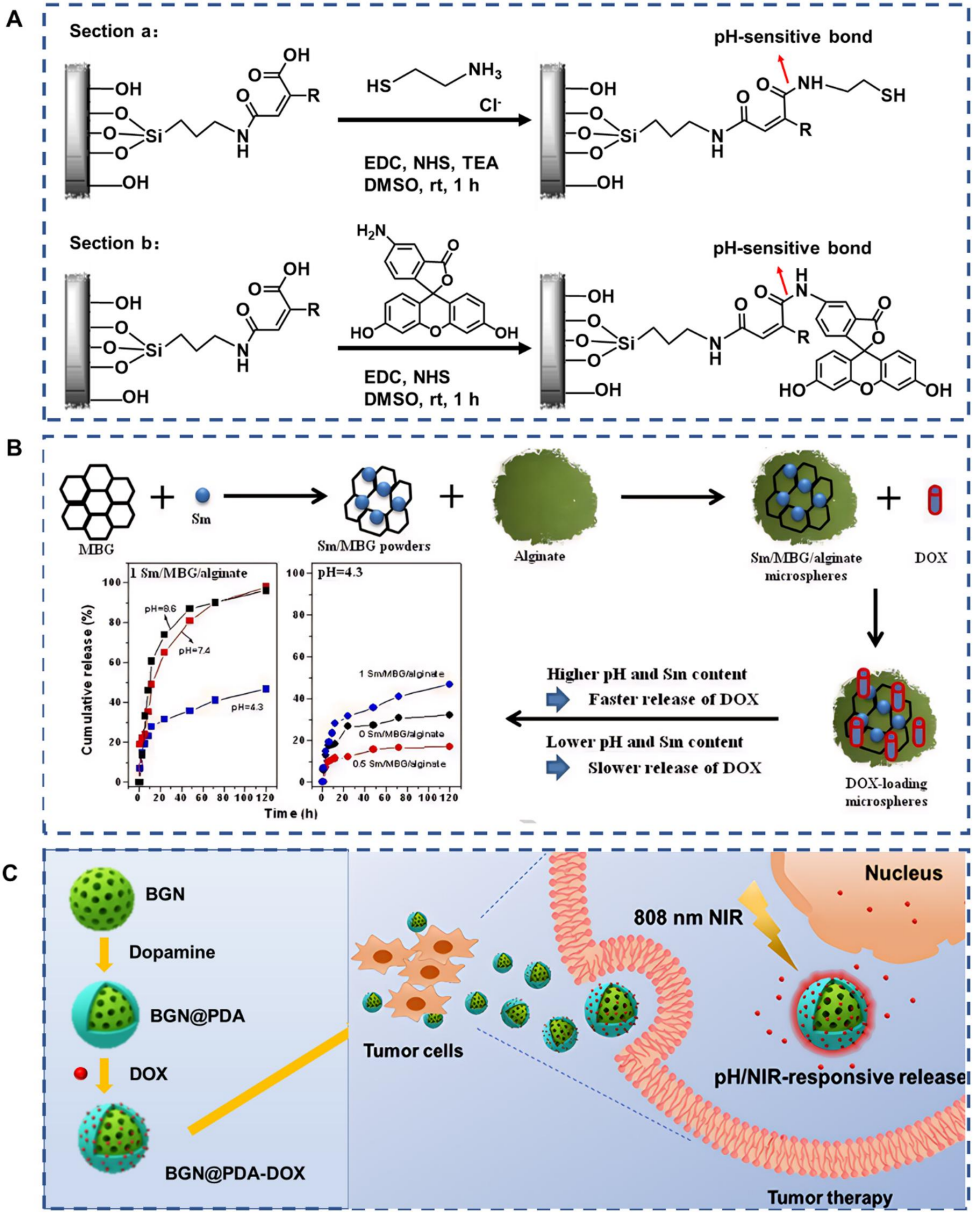
Controlled drug release of m-BGN. (**A**) Section a: Synthesis of the APTS25SG423-MA and cysteamine conjugate. Section b: Synthesis of the APTS25SG423-MA and 5-aminofluorescein conjugate [78] Copyright 2014, American Chemical Society. (**B**) The schematic illustration of controlled DOX release from the Sm/MBG alginate microspheres [88] Copyright 2016, Elsevier. (**C**) Schematic illustration of the construction of a BGN@PDA-DOX and DOX release with pH/NIR-responsive release behavior [68] Copyright 2020, American Chemical Society.

Simple passive drug delivery carrier particles lack precise treatment in complex cell microenvironments. Therefore, constructing nano-drug carriers with controllable release characteristics can not only effectively improve the drug concentration at the targeted sites and enhance the efficacy, but also reduce the side effects on non-targeted tissues and improve the safety of nano drugs. Controlled release of drugs, also known as controlled drug delivery system or drug sustained release system, refers to a drug delivery technique that changes the structure of a drug formulation through physical, chemical, or biological methods, so that the drug is automatically released from the system to the target organ or specific target tissue at a certain speed within a predetermined time, thereby maintaining the drug concentration within an effective concentration range for a long time. This technology has the advantages of fewer administration times, less fluctuation in blood drug concentration, less irritation, long-lasting efficacy, and safety and is increasingly receiving clinical attention. BGN can be used as a drug carrier to achieve controlled drug release through surface modification. After being covalently modified by organic molecular maleic anhydride, BGN was coupled with cysteamine and 5-amino fluorescein, showing that the coupling compound was completely released only under acidic (pH 4.5) conditions, but slowly released under near-neutral physiological conditions (pH 7.4). This work has opened the door for the synthesis of a series of intelligent bioactive glass [78] (Figure 13A). Composite BGN microspheres were prepared by cross-linking alginate (ALG) and Ca^2+^ to delivery of doxorubicin (DOX). The microspheres showed a continuous drug-release behavior. In addition, the delivery of DOX can be controlled by changing the concentration of the microspheres and the pH value of the microenvironment (Figure 13B). All these indicate that this material is a promising candidate for the treatment of bone cancer [88]. In addition, the polydopamine (PDA) modified BGN can achieve DOX loading, and shows an on-demand (pH/NIR response) drug release behavior, which provides a guarantee for improving the chemotherapy efficiency of tumors [68] (Figure 13C). In summary, it is known that by adding certain physical or chemical signals to the surface of BGN, BGN can respond in real-time to release drugs based on changes in the microenvironment, achieving controlled drug release.

In addition, when discussing the surface modification of BGN, it is indeed necessary to pay attention to the new properties brought by the modified materials (such as molecules, drugs, therapeutic agents or other materials), and also consider how these modifications affect the inherent properties of BGN itself, especially its ability to release therapeutic ions during dissolution. In some cases, biomolecules covering the surface of BGN and ions released from BGN may have a synergistic effect, jointly promoting a certain biological effect. For example, the ion dissolution products released by BGN (such as calcium ions and phosphorus ions) are important components of bone tissue regeneration, which can stimulate the proliferation and differentiation of bone cells, promote bone tissue repair and regeneration [92]. Therefore, when biomolecules are released simultaneously with BGN, they may work together to promote bone tissue regeneration and repair. In addition, when BGN is used as a drug carrier, the ion dissolution products it releases may alter parameters such as pH and ion strength of the local environment, thereby affecting the absorption and distribution of the drug. Meanwhile, the release of biomolecules may also have an impact on the absorption and distribution of drugs. Therefore, when biomolecules are released simultaneously with BGN, they may promote drug absorption and distribution through synergistic effects, enhancing therapeutic efficacy. In addition, the ion dissolution products released by BGN may interact with biomolecules to form stable complexes or protective layers, thereby enhancing the stability of biomolecules and prolonging their interaction time. This interaction helps to reduce the degradation and inactivation of biomolecules in the body, improving their therapeutic efficacy. In summary, the synergistic effect of biomolecule release and BGN release of ion dissolution products is a complex and interesting research field. This synergistic effect may involve the enhancement of multiple physiological processes and therapeutic effects. However, due to the complexity and diversity of the biological environment, further research and exploration are needed to determine the specific mechanisms and effects of this synergistic effect. Future research can focus on the following aspects: firstly, in-depth exploration of the interaction mechanism between biomolecules and BGN-released ion dissolution products; The second is to evaluate the impact of this synergistic effect on the therapeutic effect; The third is to develop new therapeutic methods and drug carriers based on this synergistic effect. However, the above discussion is based on the current understanding of the release characteristics of biomolecules and BGN, as well as speculations on their possible interactions within living organisms. The specific research results and conclusions may vary due to factors such as experimental conditions and material selection.

### Cell targeting

**Figure 14.**
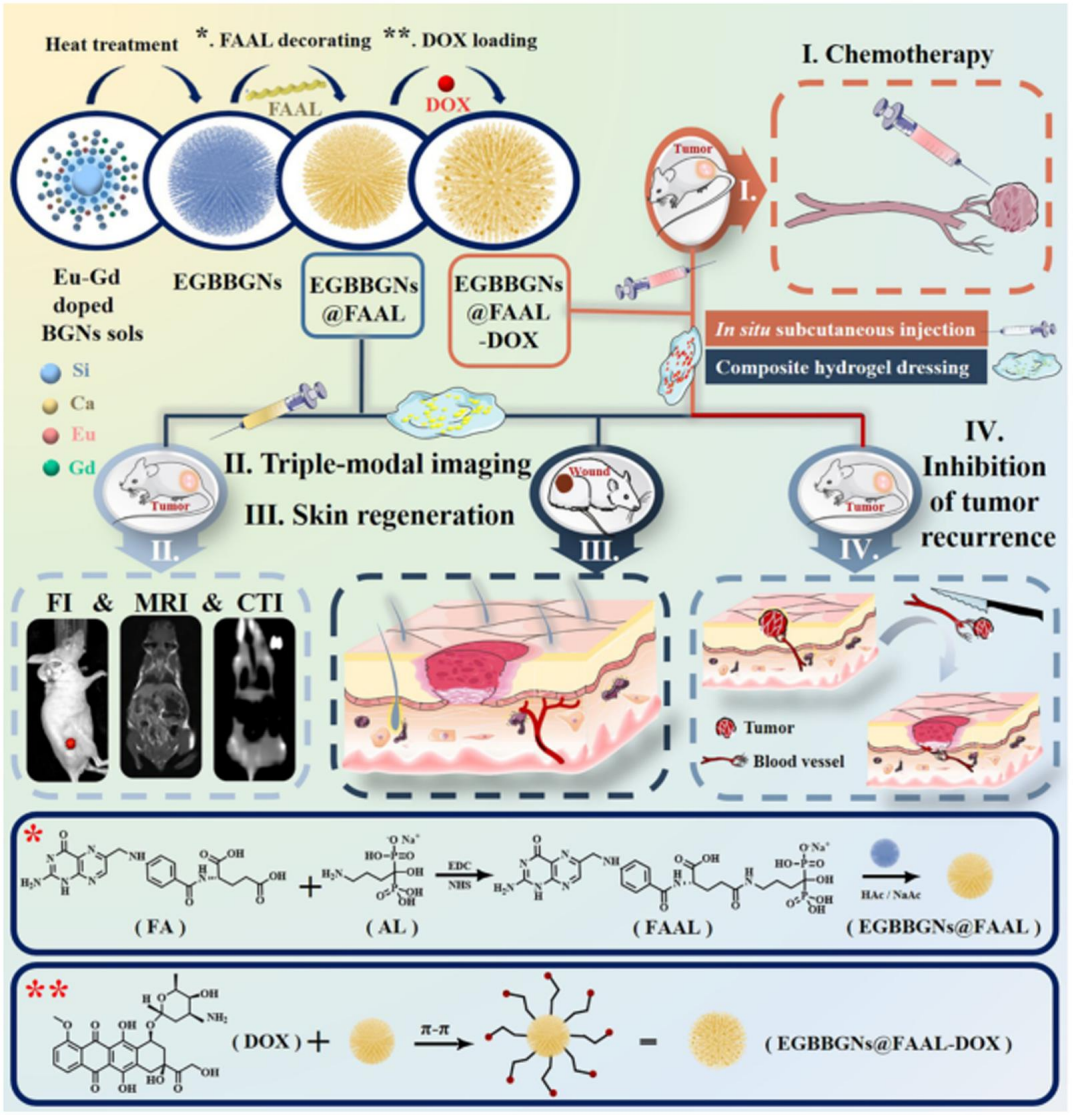
Schematic illustration describing the synthetic route of EGBBGNs@FAAL and their potentia biomedical applications for chemotherapy, triple-modal imaging, skin regeneration and the inhibition of tumor recurrence [61] Copyright 2021, Elsevier.

In the field of cancer treatment, targeted molecular design and construction is one of the research hotspots in the biomedical field. The construction of targeted drug carriers is to directly load targeted biomolecules with drugs or use the carrier's characteristics to enable chemotherapy drugs to reach and enrich in specific tissues, so it is also called ‘molecular train’. Chen *et al.* prepared a new targeted drug delivery system with folic acid (FA) as the target molecule, methotrexate (MTX) as the anti-cancer drug, and BGN as the carrier [77]. First, amino groups were grafted onto the BGN surface by APTES, and then FA and MTX were covalently coupled to the modified BGN surface by amidation reaction, thus a FA functionalized targeted drug delivery system (MTX-BGN-FA) was prepared. MTX-BGN-FA can effectively target the folate receptor of tumor cells, and can continuously release MTX *in vitro*, showing high cytotoxicity to Hela cells. In addition, the bioactive glass has excellent biological activity, and its mineralized products can also promote the healing and regeneration of damaged bone tissue. Niu *et al.* also used folic acid to graft onto the europium gadolinium doped BGN surface to achieve the targeting effect on tumor cells. Through drug loading (DOX), tumor growth can be effectively eliminated, and residual tumor cells can be removed *in situ* to inhibit local tumor recurrence, promoting the healing of the whole cortex injury wound and the wound repair caused by tumor surgery [61] (Figure 14). In addition, Chen *et al.* reported that an FBS-modified BGN targets the LAT 1 receptor, can achieve the targeting effect of tumor cells, and finally can achieve the targeted anti-tumor effect by carrying anti-cancer drugs [53]. In summary, although current research on the targeting of BGN mainly focuses on folate modification, these studies not only demonstrate the feasibility and effectiveness of BGN as a targeted drug carrier, but also lay a solid foundation for the development of more diverse and efficient tumor treatment plans in the future. With the deepening of research, BGN is expected to play a more important role in the field of tumor treatment.

### Bioimaging

**Figure 15.**
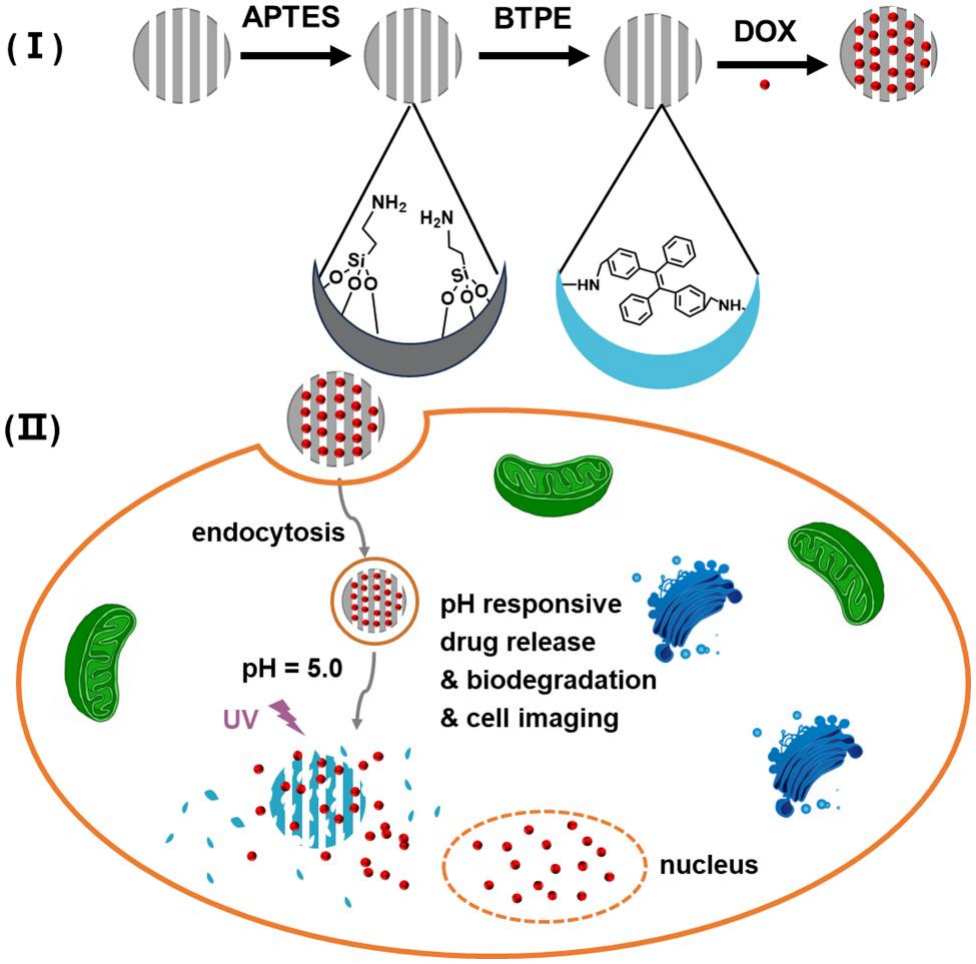
Synthesis of AIEgens-functionalized MBG and its application for drug delivery and pH-controlled drug release for cancer therapy [80] Copyright 2016, Royal Society of Chemistry.

Biomedical fluorescent labeling and probes provide a nondestructive technique for analyzing cell interactions and molecular mechanisms, and have been widely used in cell biology research and cancer cell diagnosis. When BGN is used as a drug carrier in tumor treatment, its biological imaging ability provides an essential basis for accurately detecting tumor changes. However, there are few studies on BGN bio-imaging performance for disease diagnosis. It is reported that the bioactive glass itself has certain nuclear magnetic imaging ability. It can also achieve multimodal imaging ability of BGN by doping metal ions with imaging function (such as europium ion, gadolinium ion and iron ion) [133, 134]. In addition, it is also an effective method to graft molecules with a luminescent effect on the surface. Wang *et al.* further grafted aggregation-induced luminescence molecules (AIEgens, BTPE) after the amination of BGN. The nanocomposites prepared have excellent biocompatibility and strong blue fluorescence and can be used as fluorescent probes for intracellular labeling [80] (Figure 15). The structure of 5-amino fluorescein (5-AF) contains amino groups. After the carboxylation of ABGN, 5-AF can form amide bonds with the carboxyl functional groups in ABGN, thus preparing pH-sensitive bioactive materials. Exposure to a specific pH change can promote the release of 5-AF directly at the target and achieve fluorescence detection [135]. Akbari Dourbash *et al.* developed the fifth generation of poly (amide amine)/bioactive glass inorganic-organic hybrid through direct hybridization of GPTMS as a coupling agent. The hybrid showed photoluminescence ability (emission 400–600 and 700 nm) without any organic dye or quantum dots [84]. The biological imaging capability of BGN comes from its inherent magnetic resonance imaging ability, but the signal is weak. In addition, doping ions with imaging function is also a way to improve biological imaging capability, but it has potential toxic side effects. Developing surface modification technology to modify it can effectively enhance its developing function and has the potential to achieve flexible release of fluorescent agents.

### Hemostatic property

**Figure 16.**
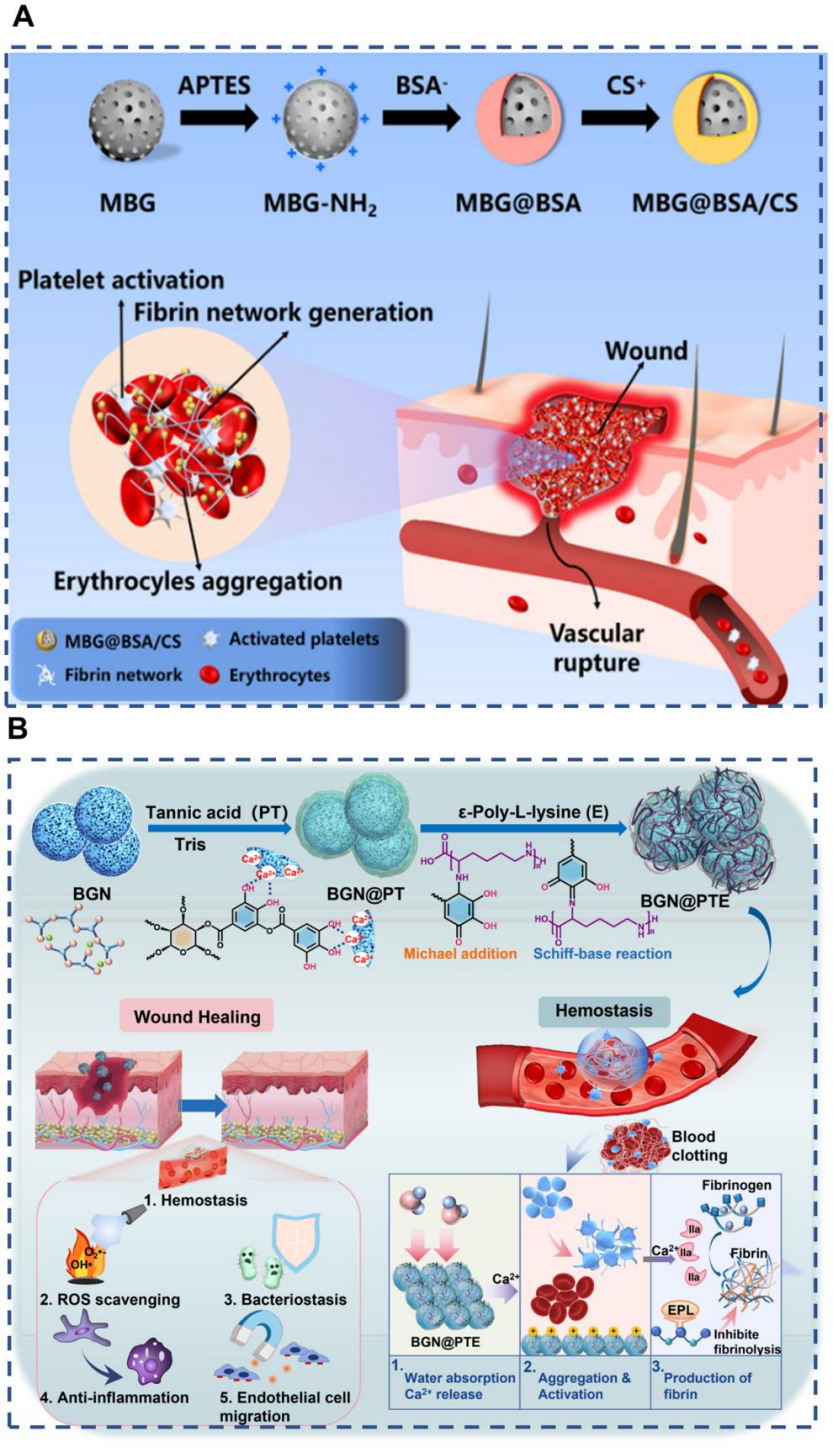
Hemostatic properties of m-BGN. (**A**) Preparation and hemostatic mechanism of MBG-based membrane-like structure camouflaged composite particles (MBG@BSA/CS) [138] Copyright 2022, Elsevier. (**B**) Illustration of the synthetic route of the multilayer-structured BGN@PTA nanosystem and excellent hemostasis functions for wound healing [119] Copyright 2022, KeAi.

The hemostasis process is the first stage of the wound healing process. When facing a large area of trauma, it is difficult to quickly achieve hemostasis, resulting in excessive blood loss and ultimately endangering human life safety. The inherent Ca^2+^ in the BGN structure has a certain hemostatic function [136, 137]. To further enhance the hemostatic ability of BGN, BGN polyelectrolyte composite (composed of carboxymethyl starch (CMS) and chitosan oligosaccharide (CS)) nanocomposites (BGN/PEC) were prepared by *in situ* coprecipitation and freeze-drying. With the addition of BGN, its degradation *in vitro* is significantly improved, and a more neutral environment is obtained, which is more suitable for surgical application. The results of plasma coagulation evaluation showed that BGN/PEC had a stronger ability to accelerate the coagulation cascade through internal pathways than PEC, but had no obvious influence on external and common pathways. The evaluation of the rabbit liver hemorrhage model showed that 10 wt% BGN/PEC had the best hemostatic effect *in vivo*. These results support the application prospect of absorbable BGN/PEC as an internal hemostatic agent, and provide a possibility for further development of a hemostatic agent based on PEC [97]. Lu group used the layer-by-layer assembly method (LBL) to coat bovine serum albumin (BSA) and chitosan (CS) on the surface of synthetic macroporous and mesoporous bioactive glass (MBG), and prepared bioactive glass-based membrane structure camouflage composite particles (MBG@BSA/CS), the composite particles can effectively activate endogenous and exogenous physiological coagulation pathways, and can immediately release Ca^2+^ to participate in the coagulation pathway, showing a significant hemostatic effect on the surface irregular bleeding (truncated model) and internal irregular deep bleeding (liver injury model) of SD rats (Figure 16A) [138]. In addition, the MBG@CMS composite microspheres were prepared by modifying the bioactive glass (MBG) to the corn starch porous microspheres (CMS) through physical adsorption, which significantly improved the water absorption, activated the internal and external coagulation pathways, and showed emergency hemostasis in the tail amputation and liver injury models of SD rats [139]. Recently, Wang *et al.* coated polytannic acid and ε-poly-L-lysine on BGN (BGN@PTE) through simple layer-by-layer assembly, and developed a multilayer structure of BGN as an integrated multilayer dressing for continuous wound management. Compared with BGN, BGN@PTE shows better hemostatic performance because it has many dependent methods to induce platelet adhesion/activation, erythrocyte blood cells (RBCs) aggregation and fibrin network formation (Figure 16B) [119]. In summary, through different modification methods and composite material designs, the hemostatic function of BGN has been significantly enhanced, opening up broader prospects for its application in wound repair. These research findings not only provide new strategies for rapid hemostasis, but also lay a solid foundation for the development of efficient and safe hemostatic materials.

## Concluding remarks and outlook

In this review, we delve into the basic development, the preparation methods and multifunctionality of the modified BGN. Si-OH is typically used as the active site of the reaction. It directly reacts with the active molecule containing -COOH, and also with various silane coupling agents through hydrolysis polymerization, introducing other active groups to the surface of BGN. BGN can serve as a reaction site for the polymerization modification of the original polymer, using its physical adsorption and ion interaction to introduce functional molecules. We also analyze the multifunctional properties of the modified BGN, such as dispersion stability, interface compatibility, antibacterial, antioxidant, anti-inflammatory, controlled drug release, targeting, bioimaging ability and hemostasis. These properties hold promise for applications in wound repair, bone tissue regeneration and tumor treatment, instilling optimism about the future of BGN.

Although the development of BGN has a history of more than 20 years, the studies on surface-functionalized BGN are relatively few. Some things could still be improved with different modification methods at the initial research stage. For example, in the covalent coupling method, Si-OH is used to hydrolyze and polymerize with various silane coupling agents, which is very dependent on the content of Si-OH on the surface of BGN. In addition, BGN is surface modified by physical adsorption effect. This method is unstable, and the surface substance will fall off within a certain time, which is not conducive to the long-term use of BGN. Therefore, developing new preparation methods has become the most important to solve the bottleneck of BGN development. Secondly, the realization of the versatility of BGN and the balance of biocompatibility needs to be further explored. Excessive introduction of functional ingredients may lead to biosafety problems. Therefore, the follow-up experimental design should balance the relationship between various properties, hoping to optimize BGN performance. In addition, the modified BGN is mainly focused on tumor treatment. However, there has yet to be a breakthrough in the application of tumor post-operative wounds, and little work has been done on using modified BGN for tissue regeneration. Therefore, expanding the application of m-BGN in tissue regeneration, including tumor treatment, wound repair, and bone tissue repair, has become one of the important research directions of modified BGN. Finally, after the functionalization of BGN, modified BGN can be obtained, which can be combined with other substrates, including hydrogel, degradable metal, polymer elastomer, etc. to prepare multifunctional composite materials, further realizing the broad application of m-BGN.

## Supplementary Material

rbae110_Supplementary_Data
