# Supplementary material for: Multifunctional bioactive glass nanoparticles: surface–interface decoration and biomedical applications
Source: Regen Biomater. 2024 Sep 6;11:rbae110. doi: 10.1093/rb/rbae110 (PMC11422188; doi:10.1093/rb/rbae110)

**
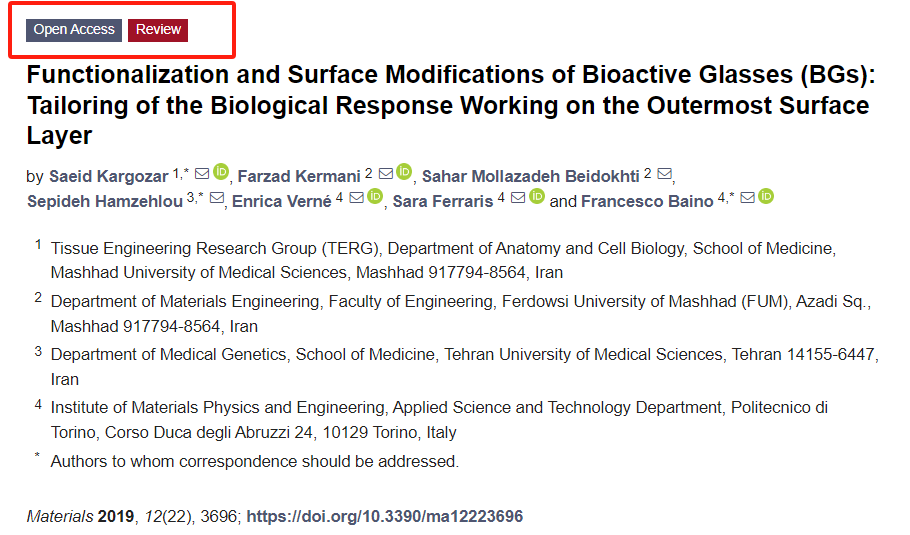
Figure 3:** [73]


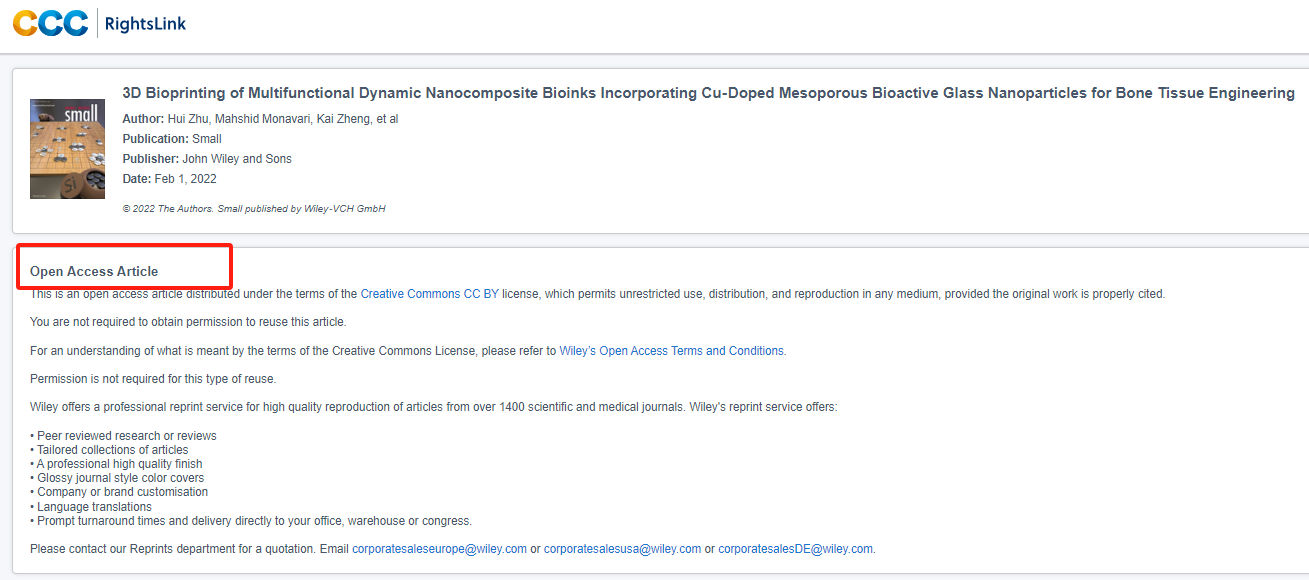
 **Figure 4:** [75]

**Figure 4:** [77]


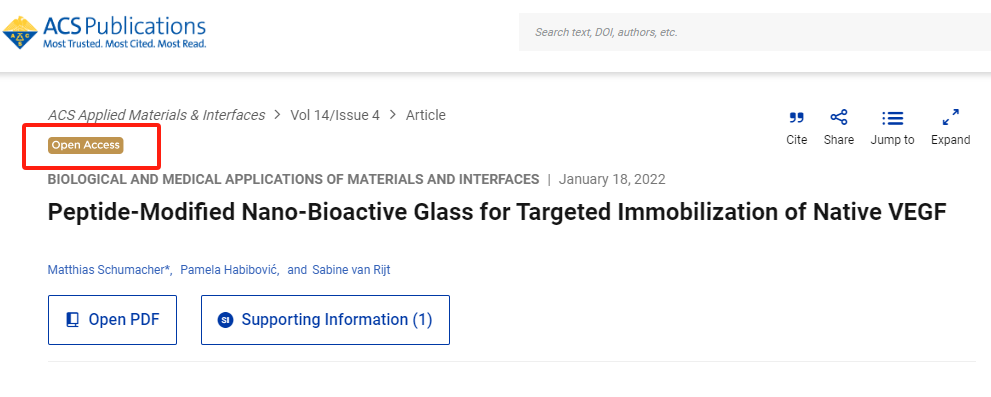


**Figure 4:** [80]


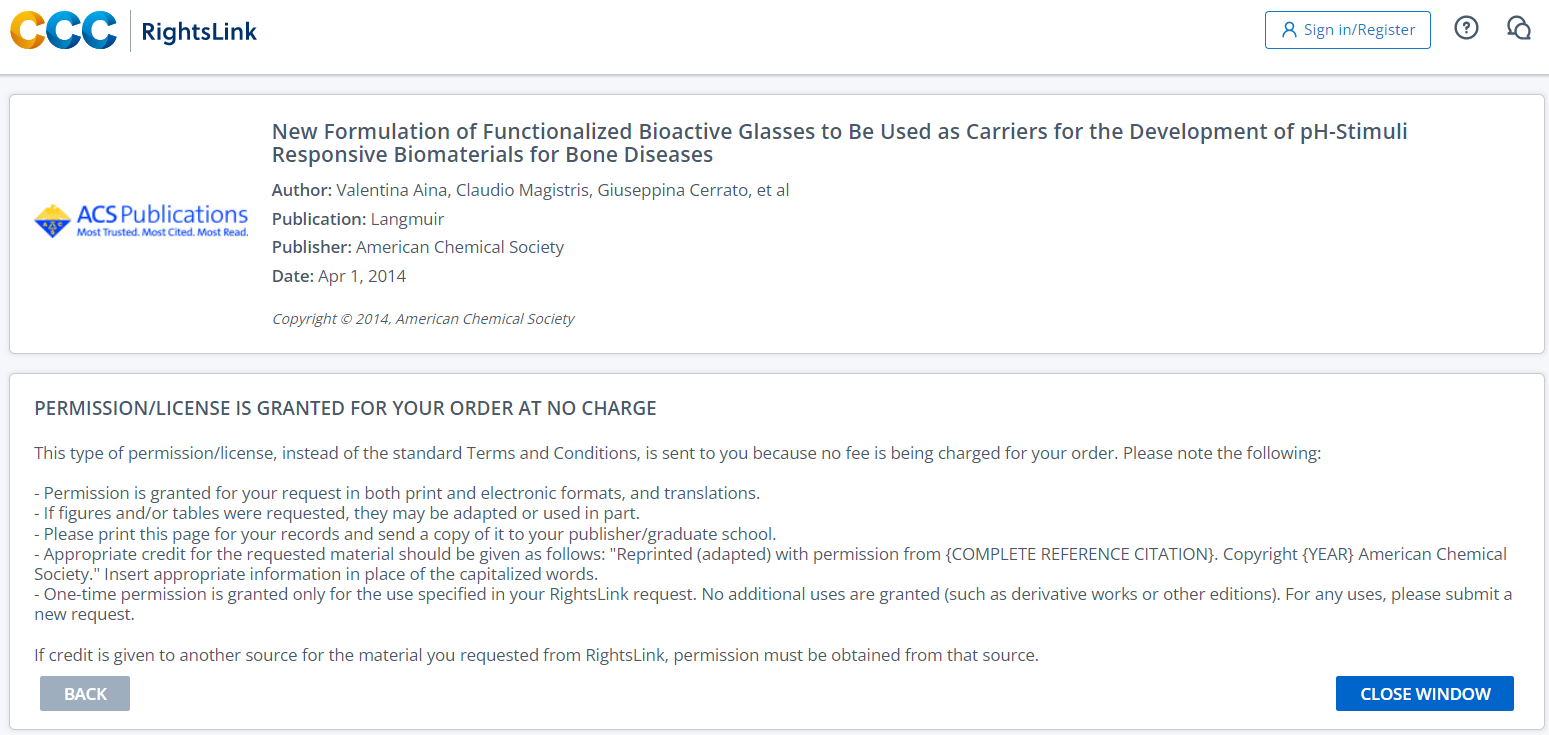


**Figure 4:** [82]


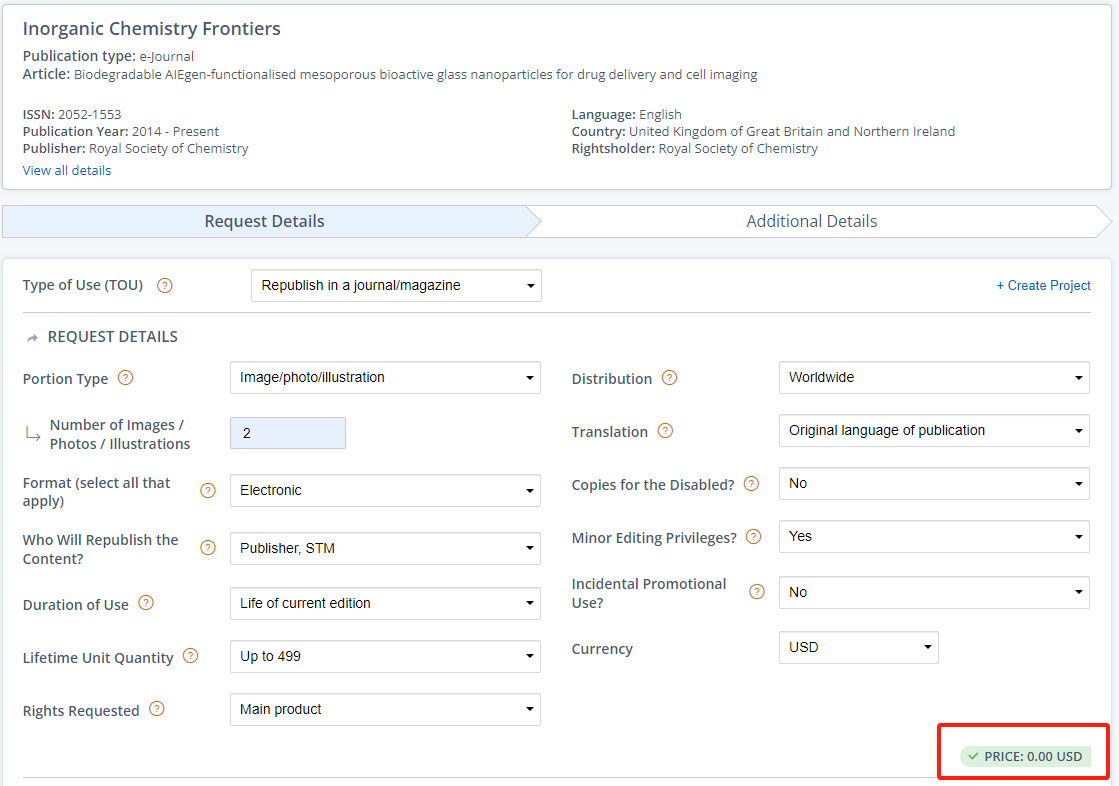


**Figure 6:**
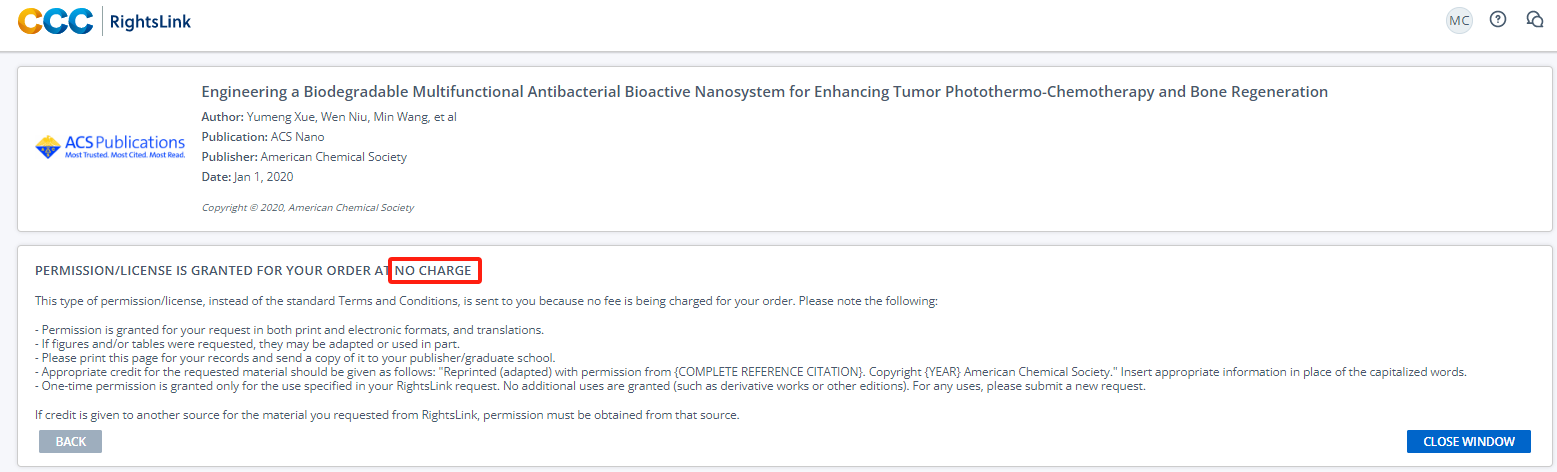
[70]

**Figure 6:** [120]


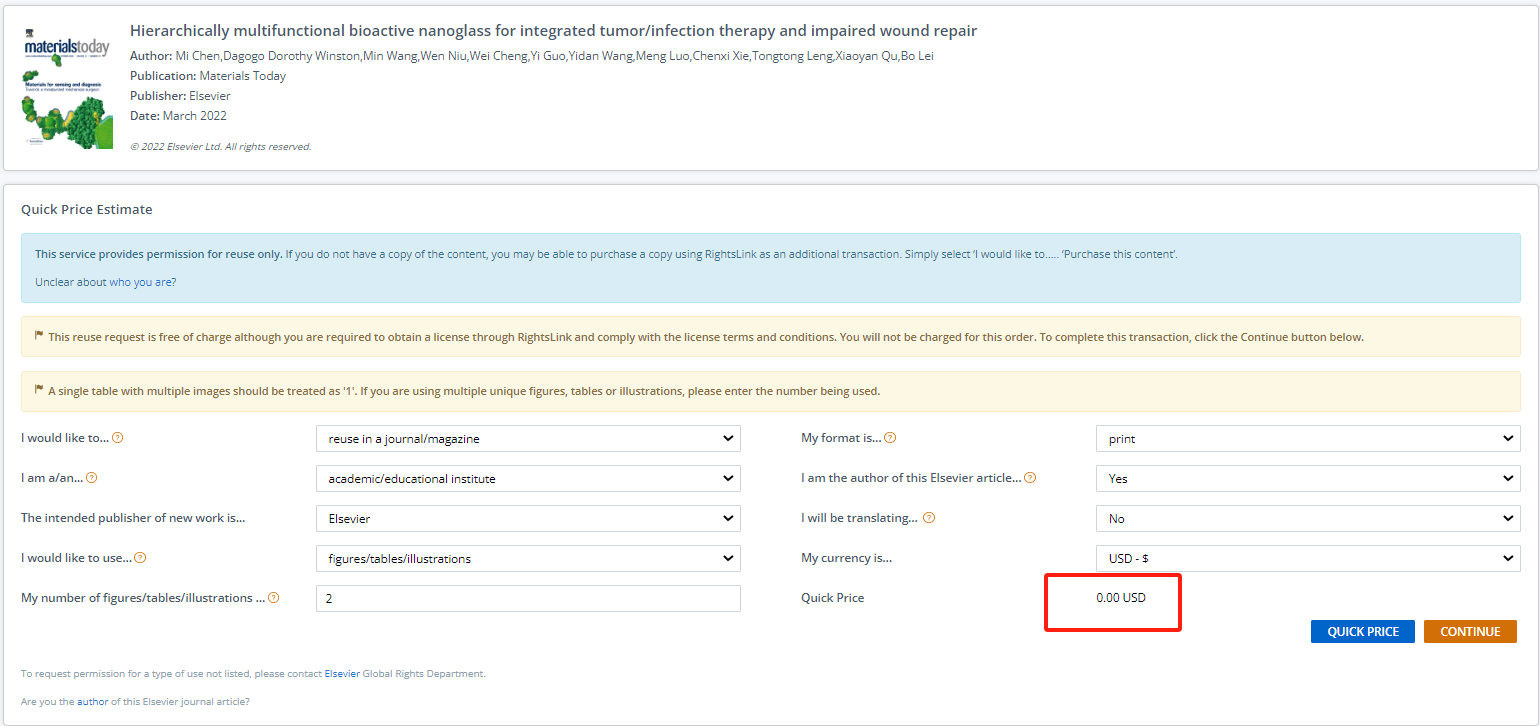


**Figure 6:** [121]


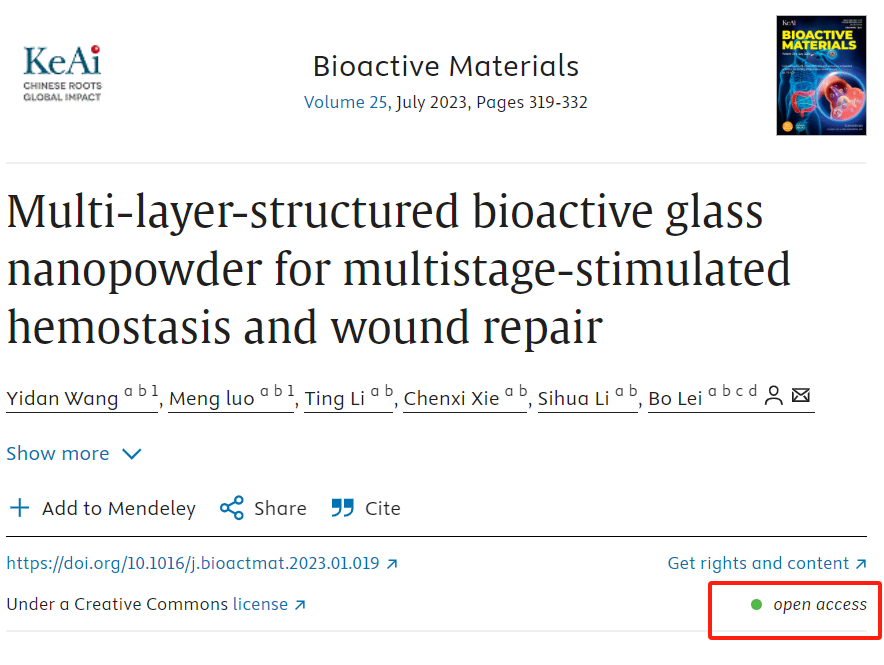


**Figure 7:** [97]


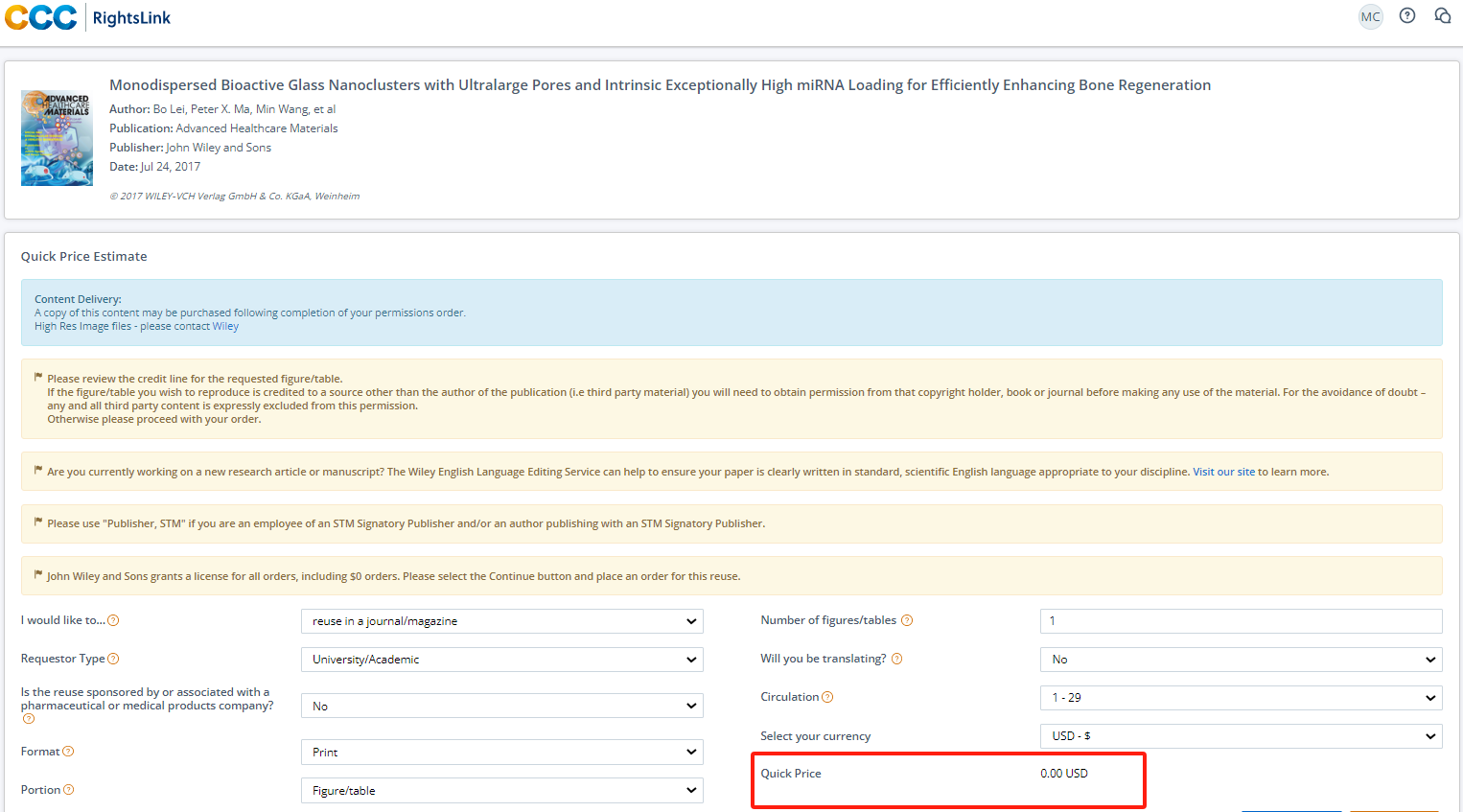


**Figure 7:** [98]


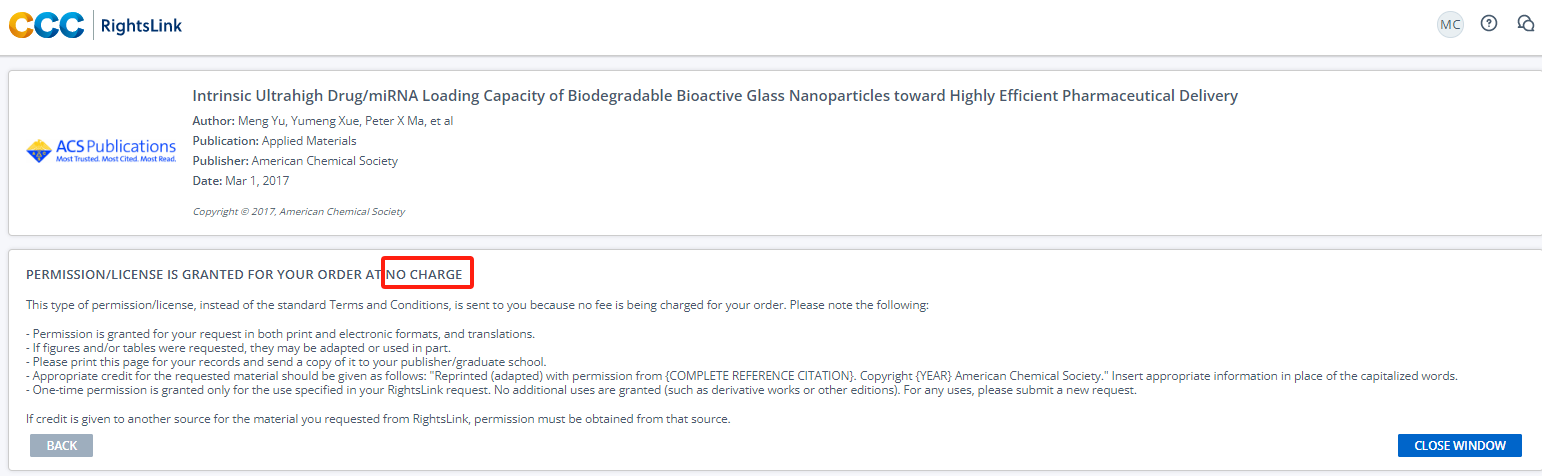


**Figure 8:** [68]


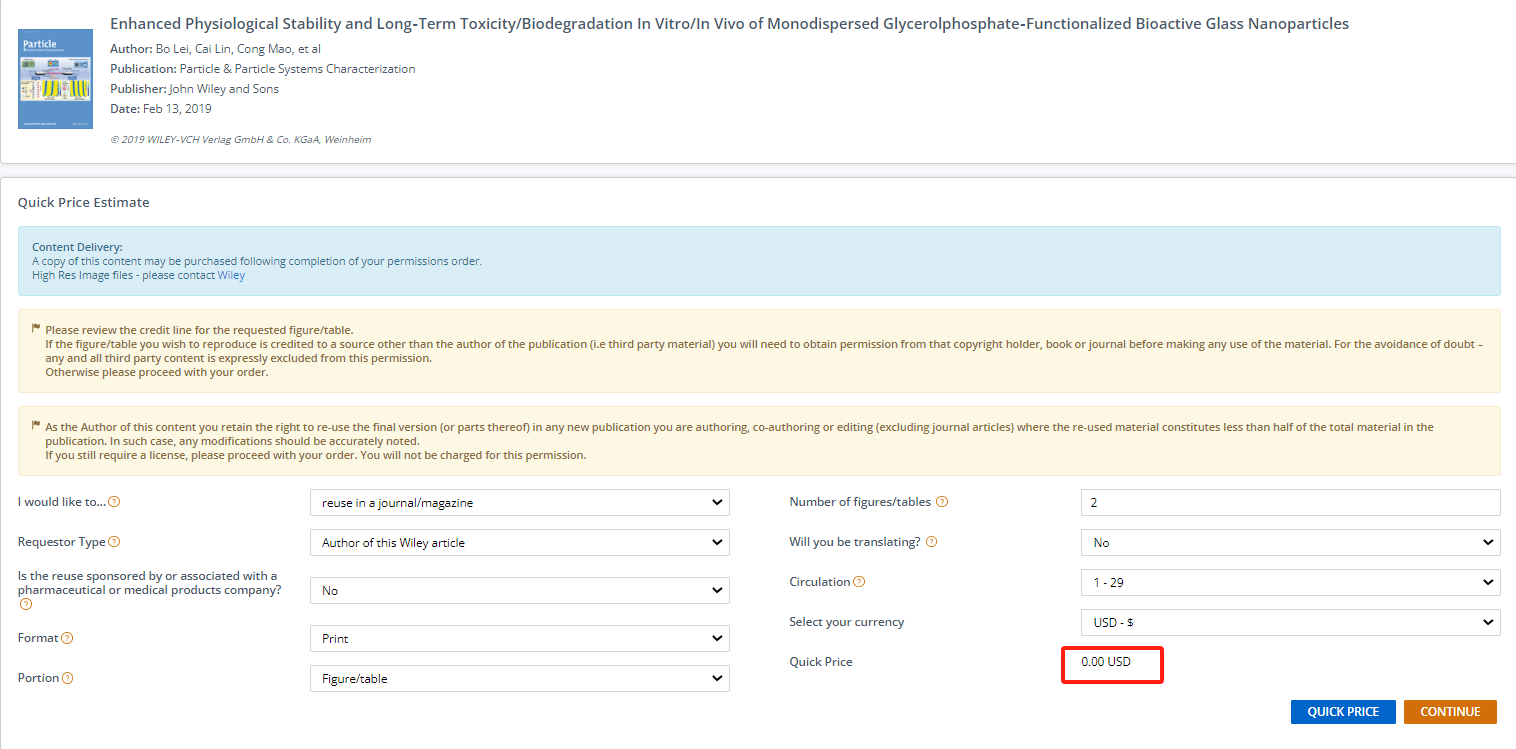


**Figure 8:** [53]


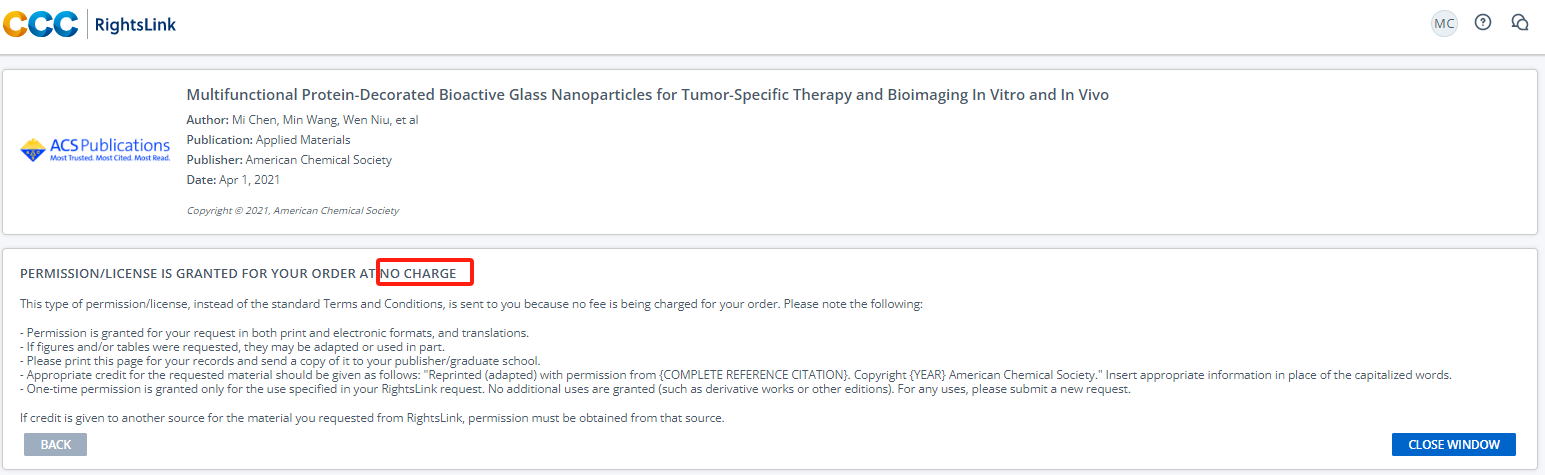


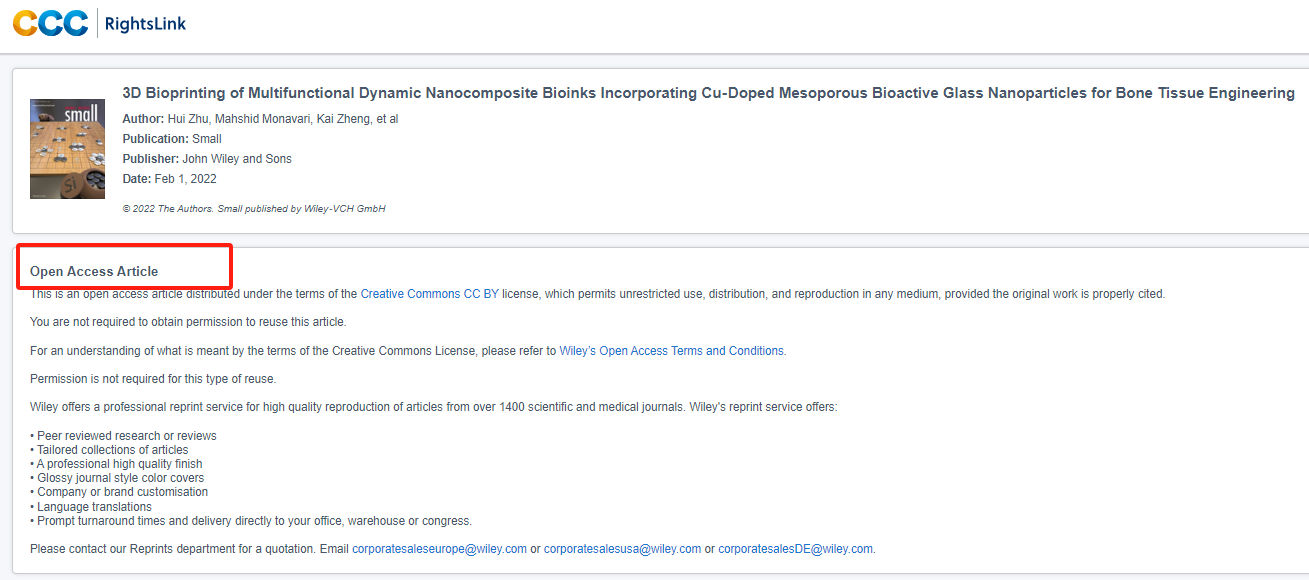
 **Figure 9:** [75]

**Figure 10:** [123]


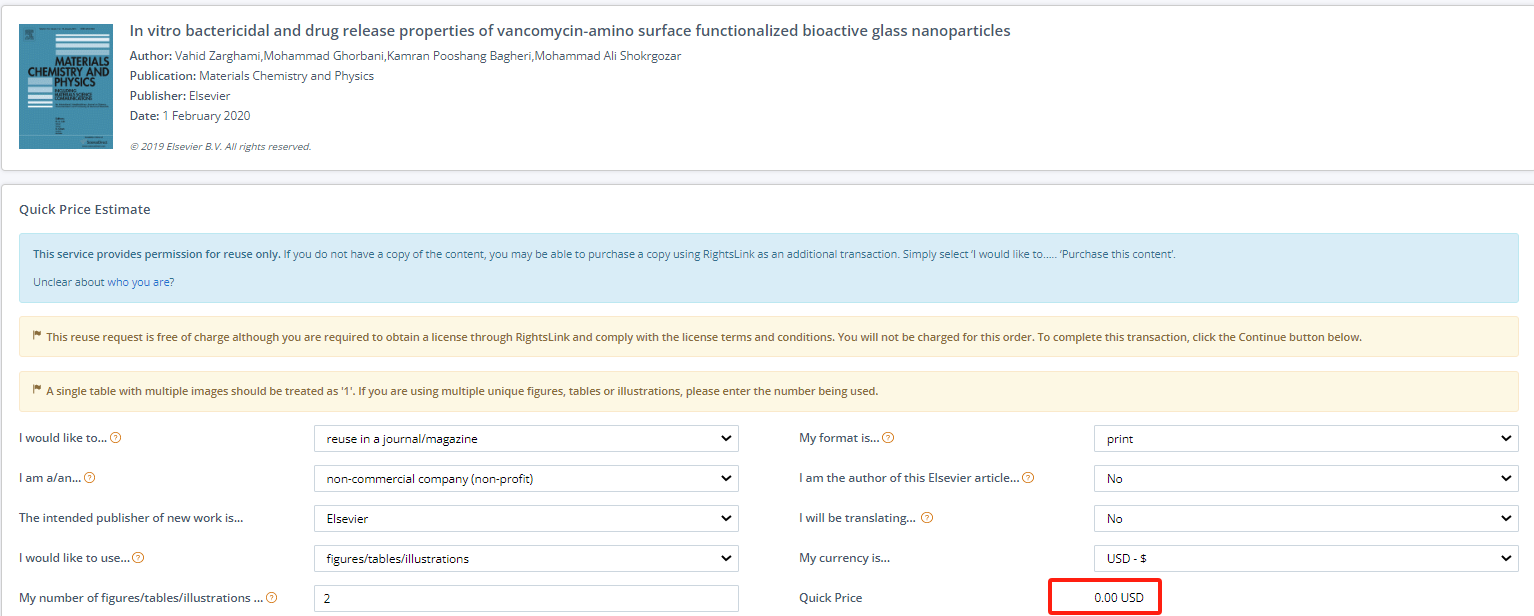


**Figure 10:**
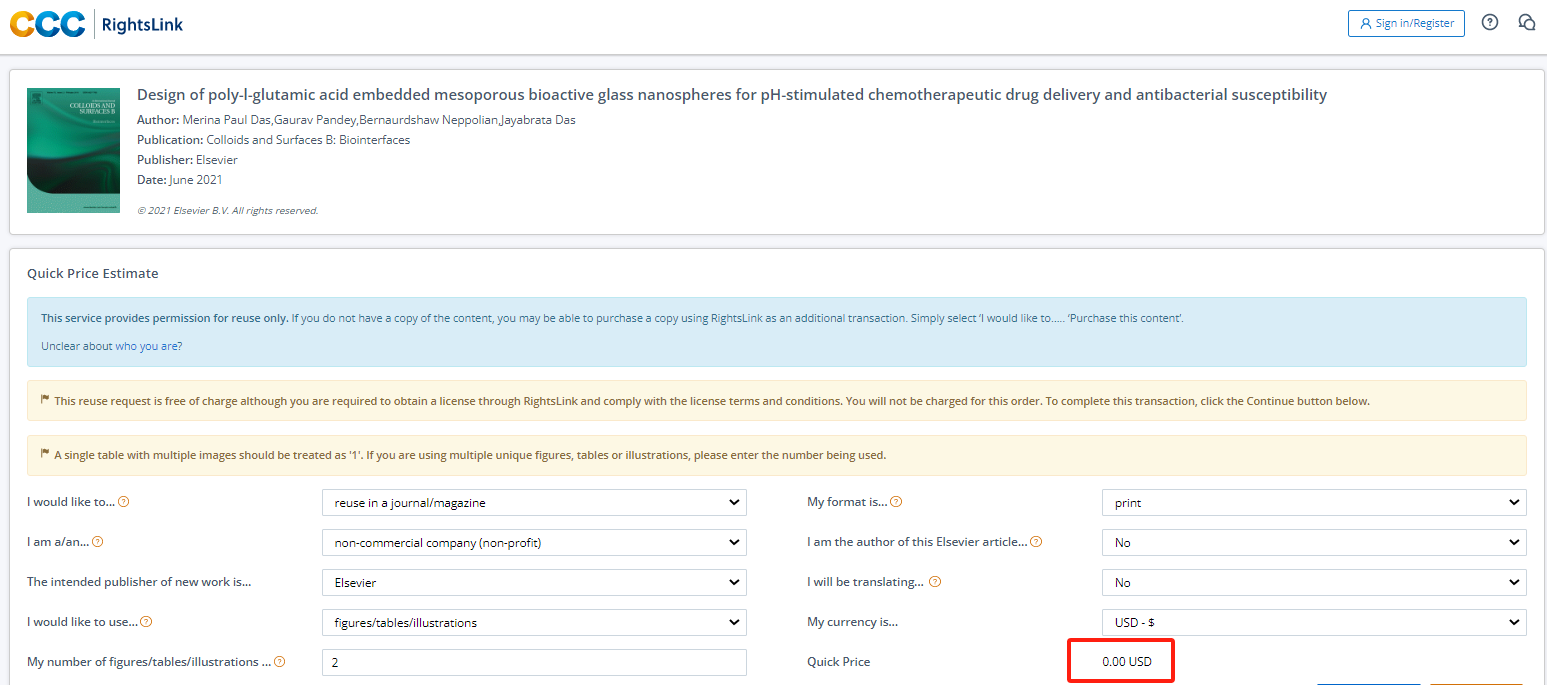
[124]


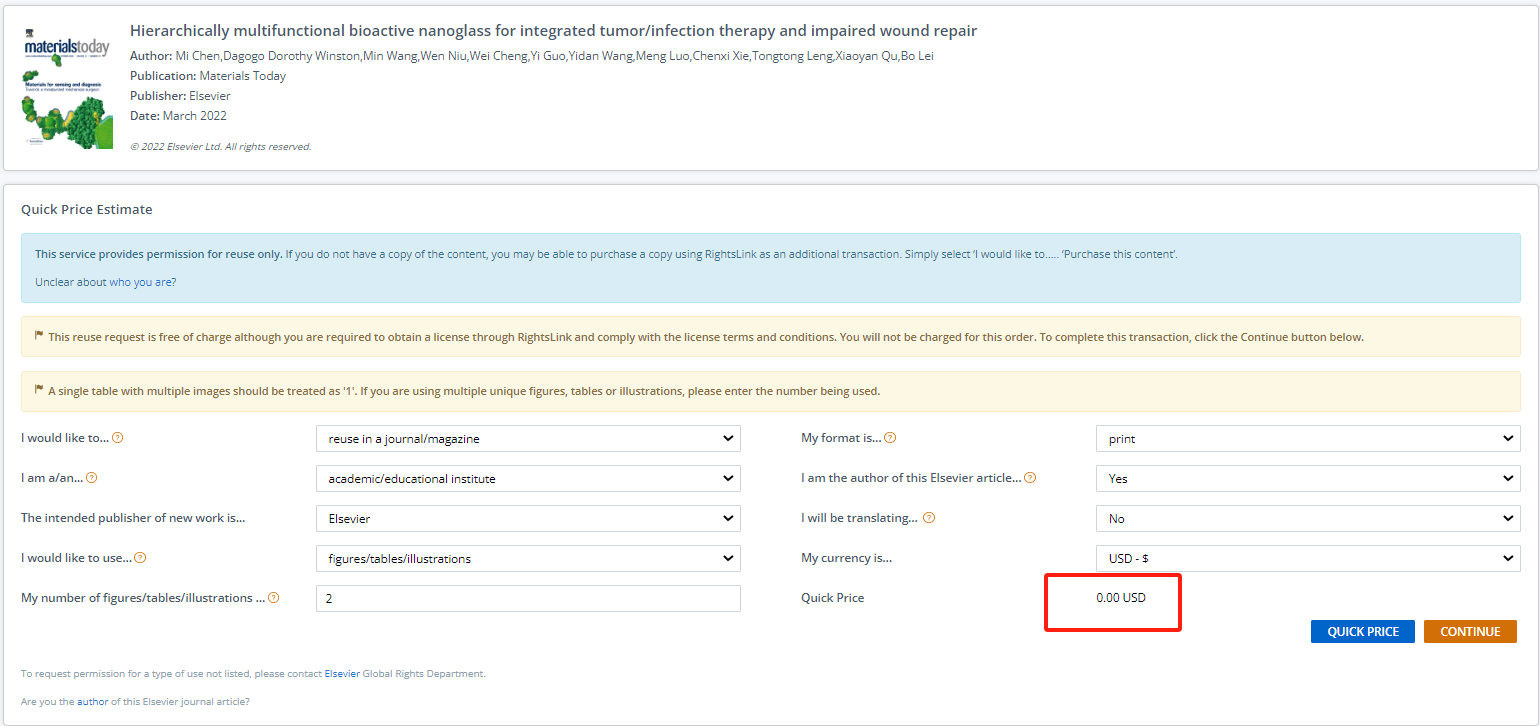
 **Figure 10:** [120]

**Figure 11:**
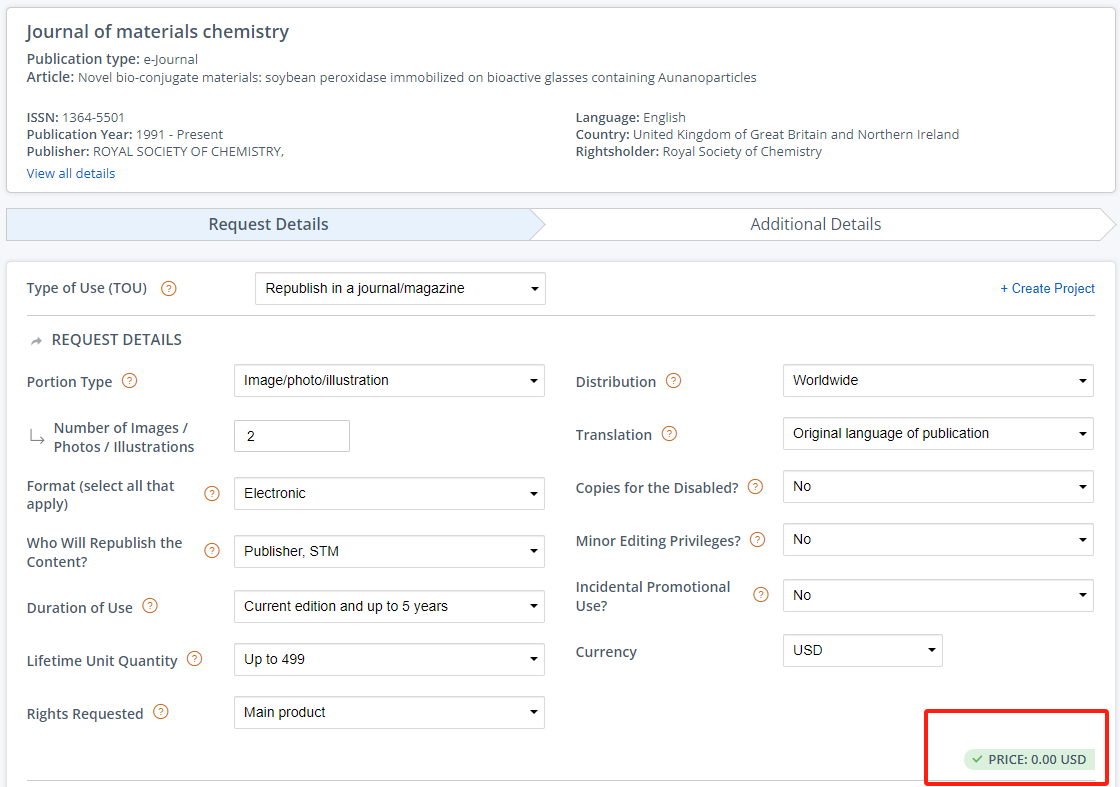
 [88]

**Figure 11:** [118]


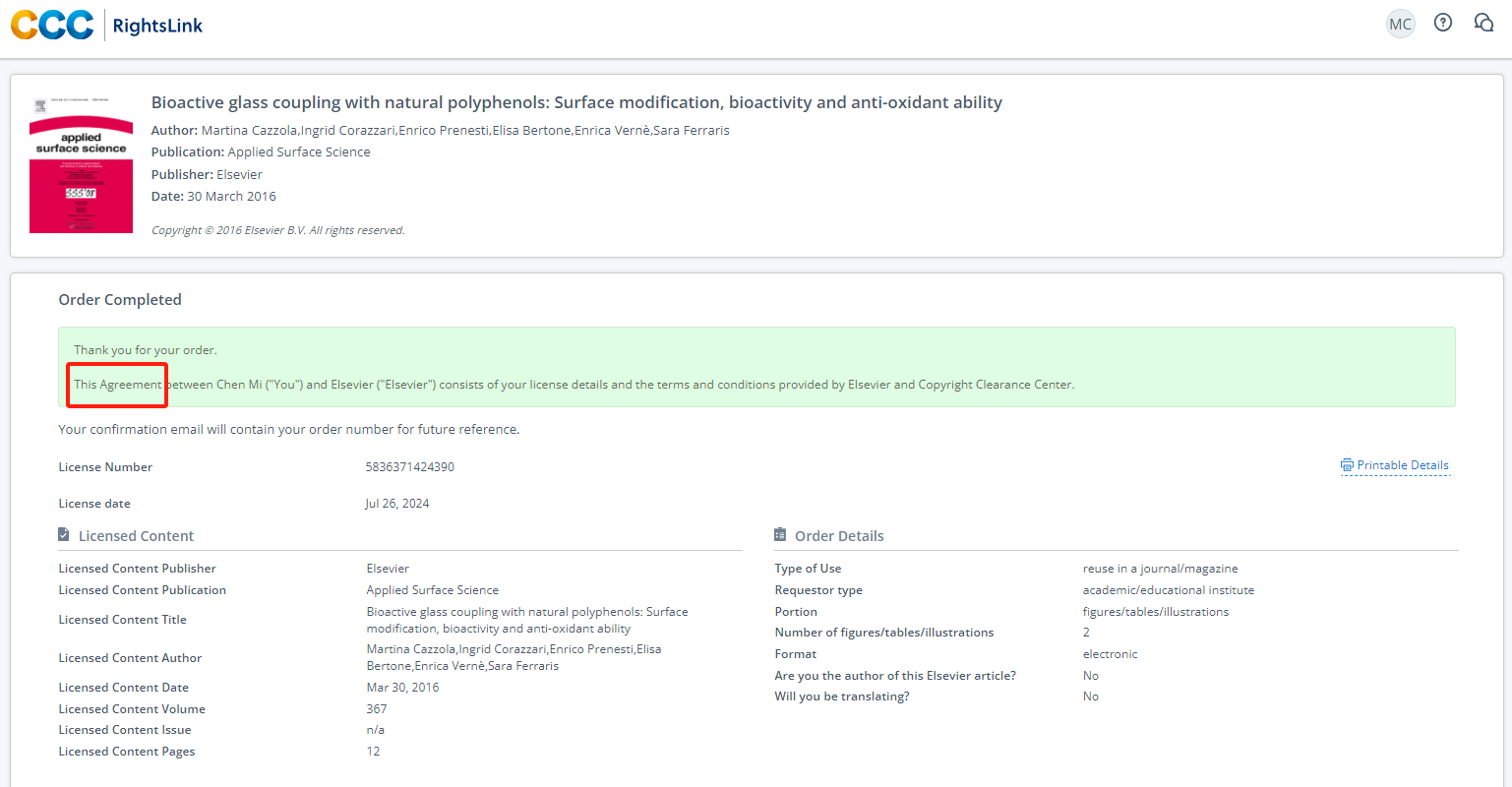


**Figure 12:**
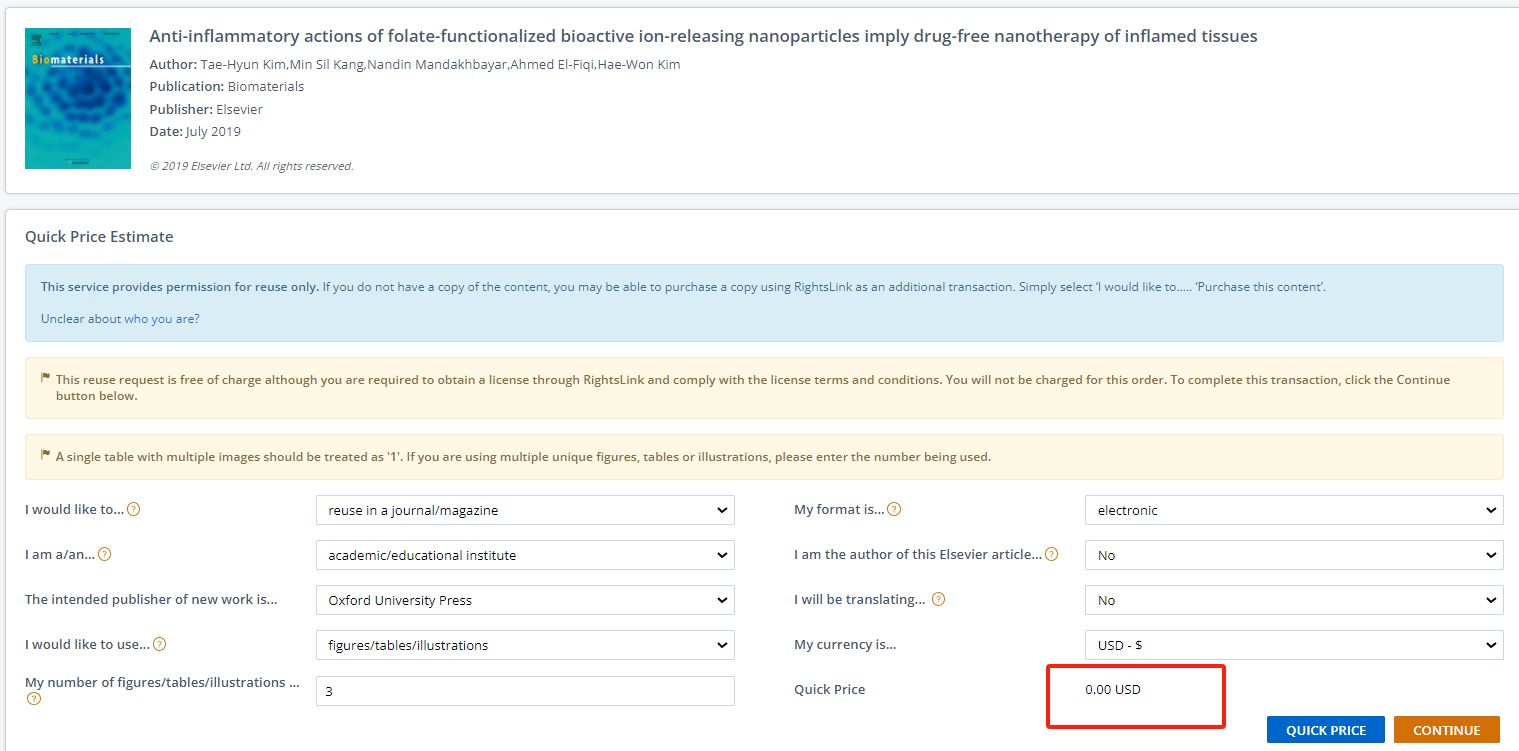
 [134]

**Figure 13:** [80]


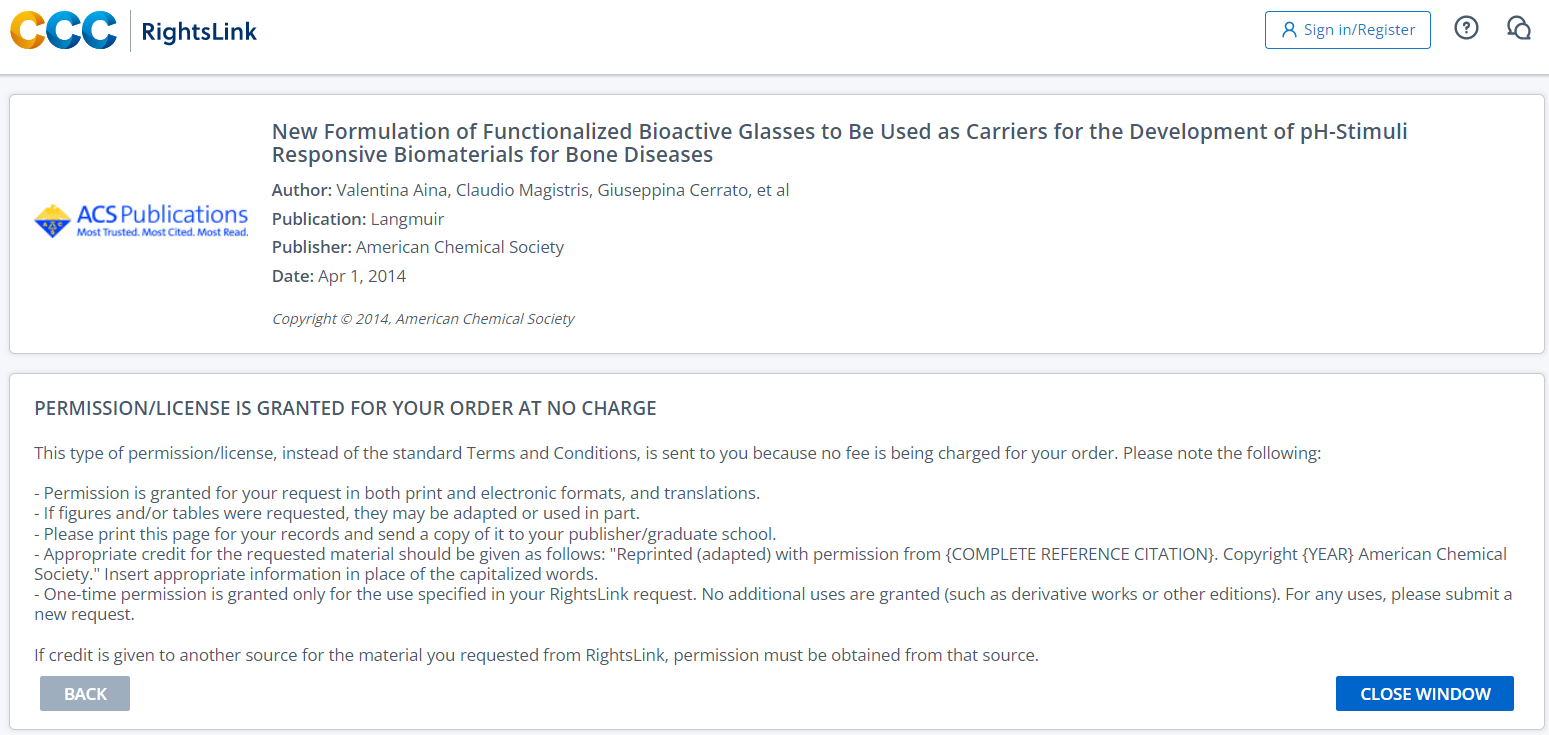


**Figure 13:** [90]


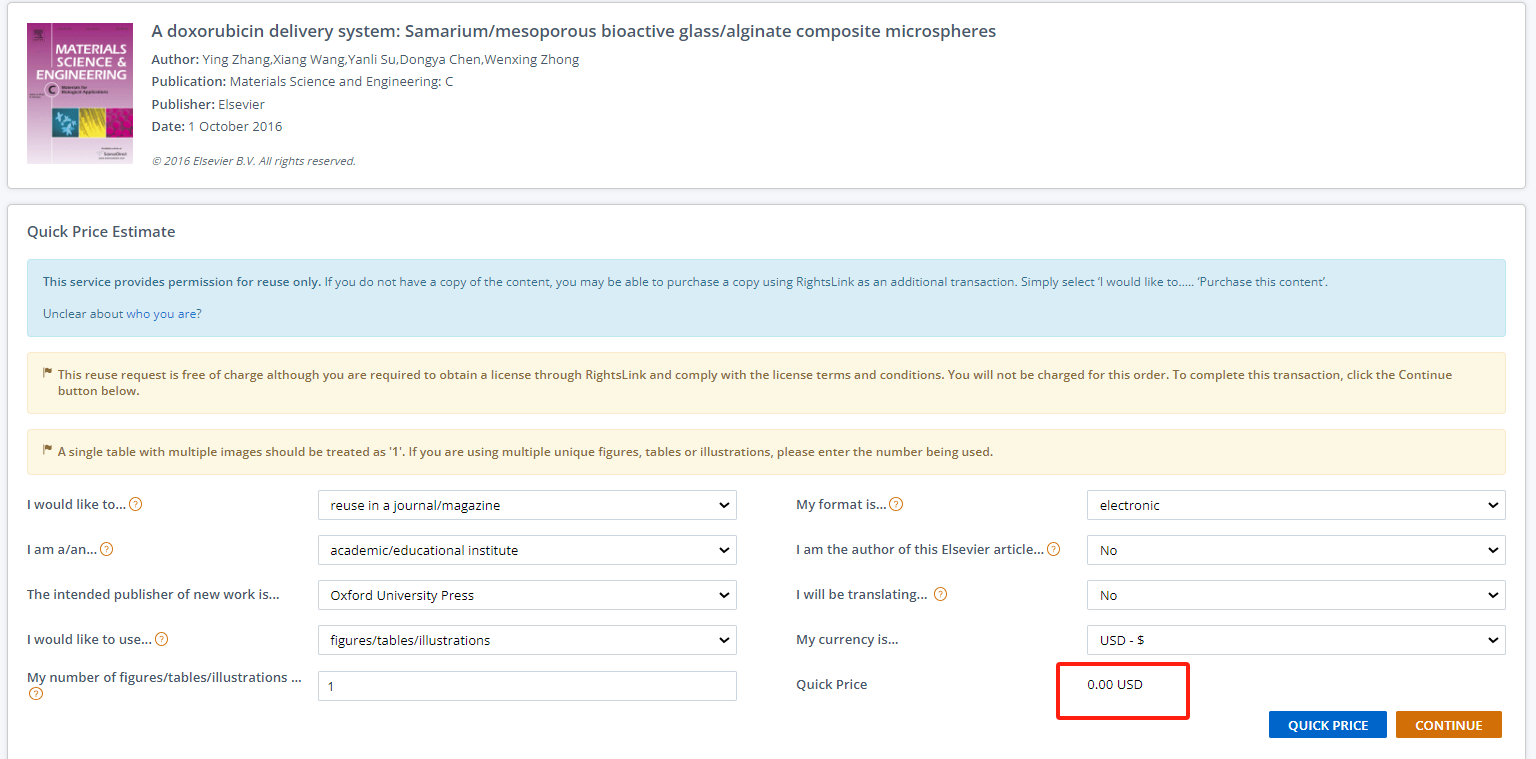


**Figure 13: [**70]


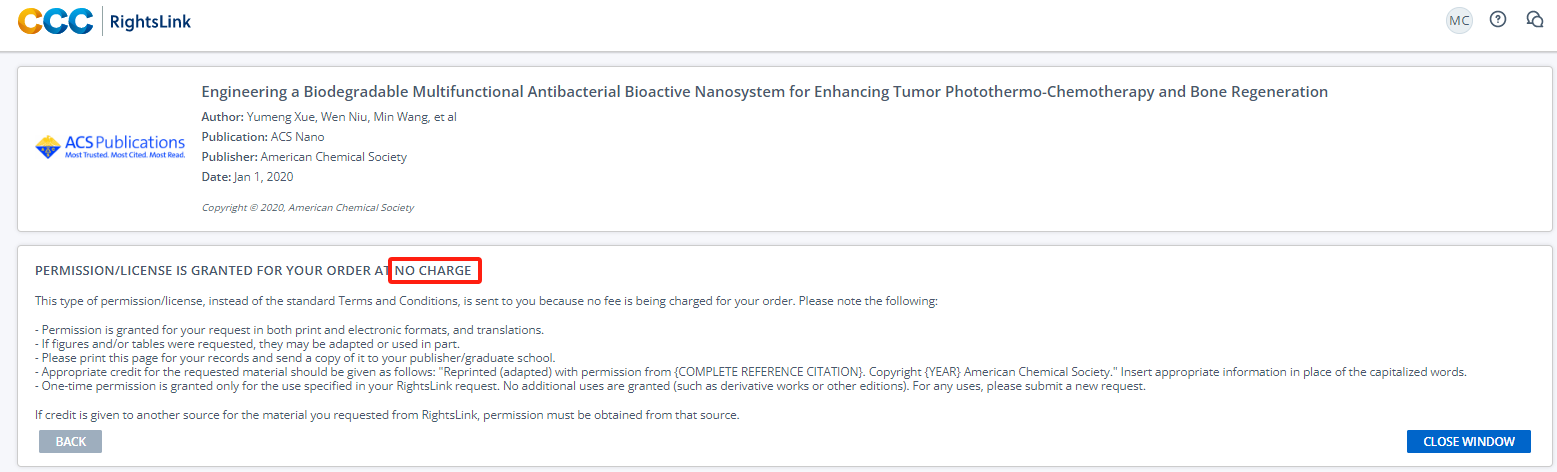


**Figure 14:** [62]

**Figure 15:**
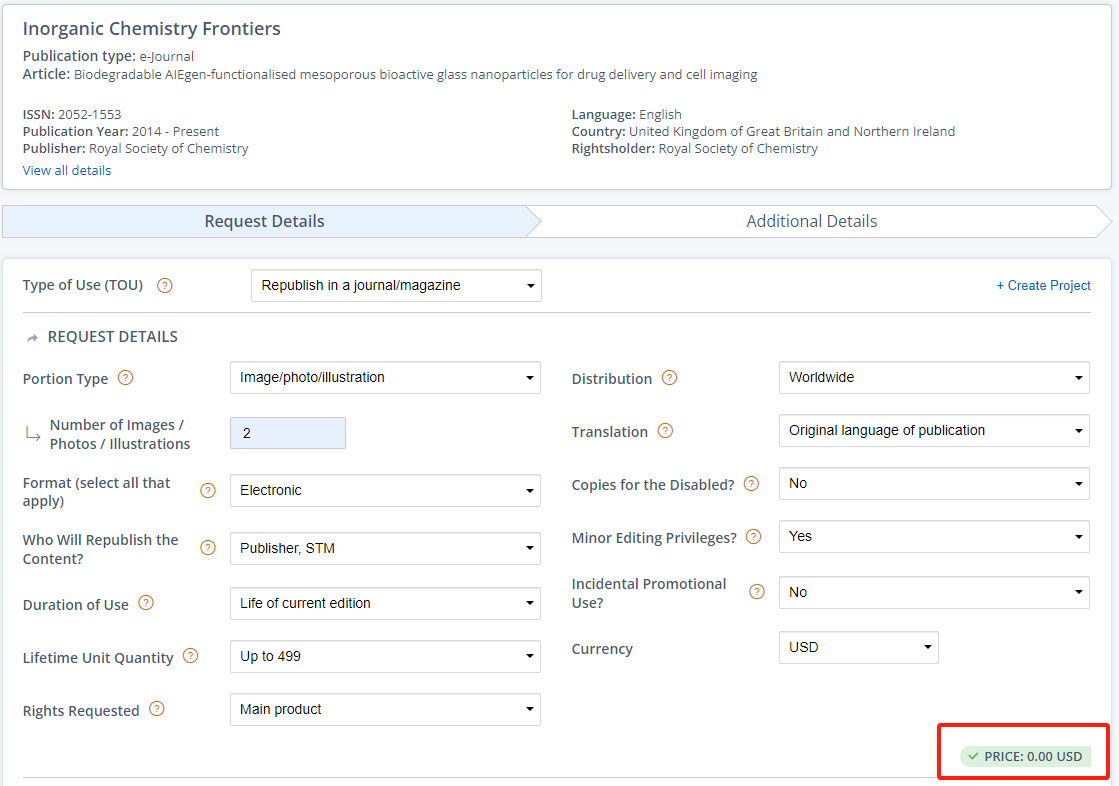

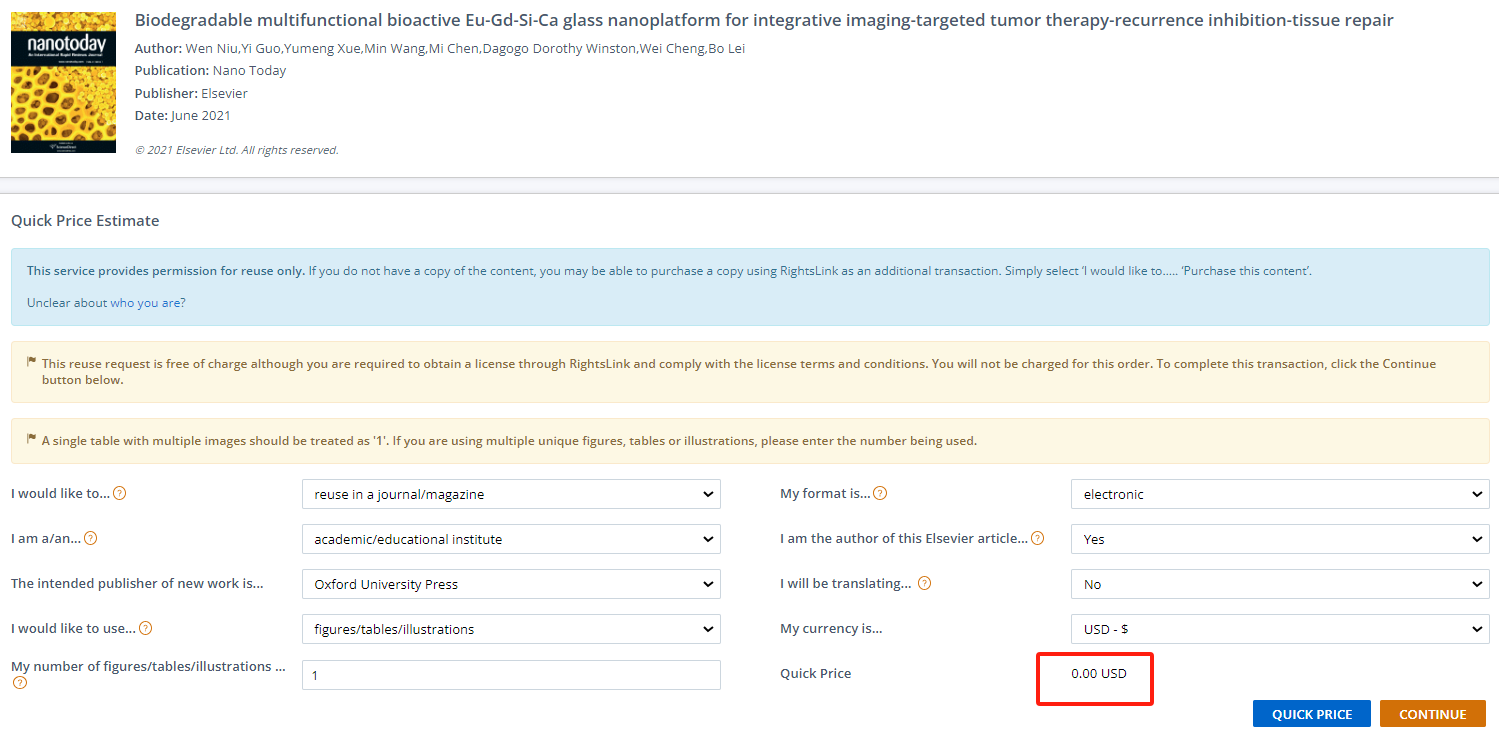
 [82]

**Figure 16:** [140]

**Figure 16:**
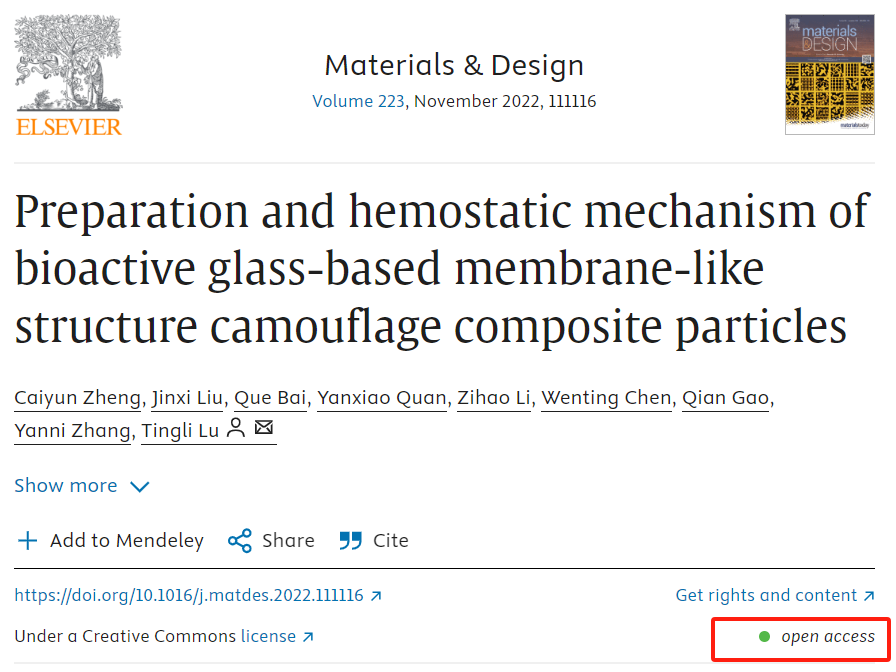
[121]


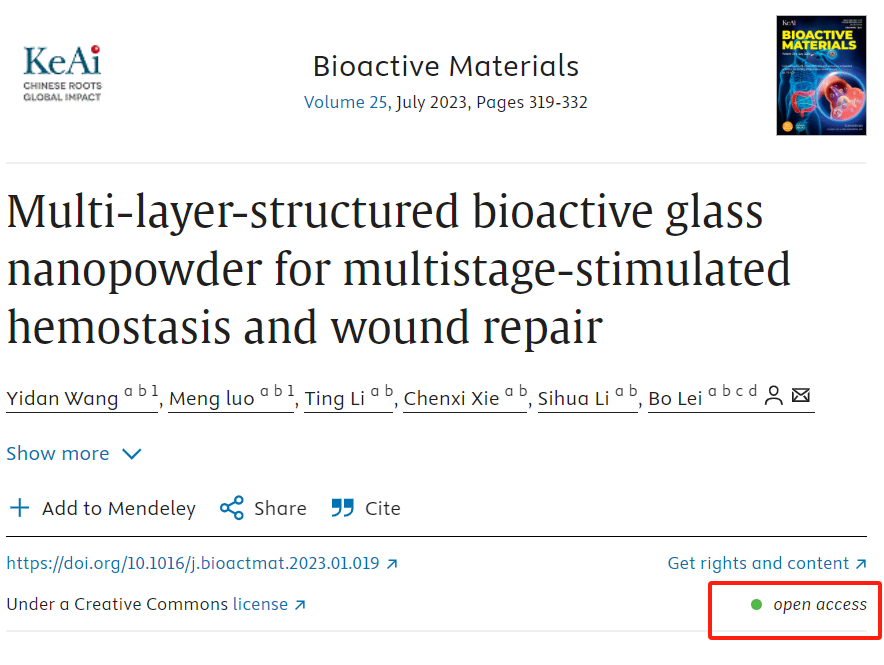

Supplement: rbae110_Supplementary_Data [file rbae110_supplementary_data.docx]
